# Acute muscle mass loss was alleviated with HMGB1 neutralizing antibody treatment in severe burned rats

**DOI:** 10.1038/s41598-023-37476-4

**Published:** 2023-06-24

**Authors:** Juquan Song, Imran H. Chowdhury, Subhadip Choudhuri, Amina E. I. Ayadi, Lizette E. Rios, Steven E. Wolf, Joseph C. Wenke, Nisha J. Garg

**Affiliations:** 1 Department of Surgery, University of Texas Medical Branch, Galveston, TX USA; 2 Department of Microbiology and Immunology, University of Texas Medical Branch, Galveston, TX USA; 3 Department of Orthopedic Surgery and Rehabilitation, University of Texas Medical Branch, Galveston, TX USA; 4 Institute for Human Infections and Immunity, University of Texas Medical Branch, Galveston, TX USA

**Keywords:** Trauma, Translational research, Acute inflammation, Skeletal muscle, Acute inflammation, Molecular medicine, Experimental models of disease, Mechanisms of disease

## Abstract

Burn injury is associated with muscle wasting, though the involved signaling mechanisms are not well understood. In this study, we aimed to examine the role of high mobility group box 1 (HMGB1) in signaling hyper-inflammation and consequent skeletal muscle impairment after burn. Sprague Dawley rats were randomly assigned into three groups: (1) sham burn, (2) burn, (3) burn/treatment. Animals in group 2 and group 3 received scald burn on 30% of total body surface area (TBSA) and immediately treated with chicken IgY and anti-HMGB1 antibody, respectively. Muscle tissues and other samples were collected at 3-days after burn. Body mass and wet/dry weights of the hind limb muscles (total and individually) were substantially decreased in burn rats. Acute burn provoked the mitochondrial stress and cell death and enhanced the protein ubiquitination and LC3A/B levels that are involved in protein degradation in muscle tissues. Further, an increase in muscle inflammatory infiltrate associated with increased differentiation, maturation and proinflammatory activation of bone marrow myeloid cells and αβ CD4^+^ T and γδ T lymphocytes was noted in in circulation and spleen of burn rats. Treatment with one dose of HMGB1 neutralizing antibody reduced the burn wound size and preserved the wet/dry weights of the hind limb muscles associated with a control in the markers of cell death and autophagy pathways in burn rats. Further, anti-HMGB1 antibody inhibited the myeloid and T cells inflammatory activation and subsequent dysregulated inflammatory infiltrate in the muscle tissues of burn rats. We conclude that neutralization of HMGB1-dependent proteolytic and inflammatory responses has potential beneficial effects in preventing the muscle loss after severe burn injury.

## Introduction

Severe burns are a common injury with an occurrence rate of 5/100,000 people per year globally^1^. American Burn Association documented that, 205,033 burn patients received inpatient treatment in in the United States in year 2015. In patients with burn on ≥ 30% of the TBSA, significant adverse effects on the organs function are noted^2^. Severely burned patients may exhibit a loss of > 25% of the total body mass acutely that can continue for more than two years after burn injury^3,4^. Muscle loss also increases the risk of death after injury, such that a 40% loss of lean body mass carries a 90% mortality risk^5^. Increased understanding of the pathogenic mechanisms of muscle loss would lead to the development of needed new treatments for improving the muscle mass and function and improve the quality of life in burn patients.

Muscle wasting is presented as a cachexic state with breakdown of skeletal muscle and adipose tissue. Cachexia occurs even when skeletal muscle is active and loaded, and it is routinely observed in burn patients^6^. Recent transcriptomic studies suggest that systemic inflammatory response associated with protein hyper-catabolism and mitochondrial metabolic dysfunction are the hallmarks of muscle wasting in burn injury^7^. Indeed, elevated serum levels of inflammatory cytokines, including tumor necrosis factor (TNF)-α and interleukin (IL)-6, were noted in severe burn patients^8^. Our in vitro studies showed TNF-α inhibited myogenesis and IL-6 stimulated mitochondrial fragmentation after burn^9,10^. Up regulation of ubiquitin-proteolytic pathway along with mitochondrial dysfunction and inflammation are also noted in cachexia of diverse etiologies^11–13^. Hyper-activated immune response and sepsis post-trauma are associated with multiple organ dysfunction^14^, and it is likely that inflammation also contributes to muscle wasting in trauma and other debilitating chronic diseases. Key mechanisms that drive inflammation and downstream mechanisms of muscle loss in burn injury remain to be elucidated.

High mobility group box protein 1 (HMGB1) is a DNA-binding nuclear protein that maintains nucleosome structure, and DNA replication and repair^15^. In stressed conditions, HMGB1 is released by activated immune cells and necrotic cells into the extracellular milieu^16^. Secreted HMGB1 potentiates cellular exhaustion, impaired healing, and tissue necrosis^17^. Systemic increases in HMGB1 levels and its association with poor survival outcomes were noted in burn patients^18^. In collagen-induced arthritis model, administration of HMGB1 neutralizing antibody (Ab) significantly ameliorated the weight loss and cartilage destruction in arthritic mice^19^. A recent study showed that expression and serum levels of HMGB1 were increased in rats exposed to trauma-induced severe injury, and neutralizing systemic HMGB1 improved the fracture healing through modulating γ∂ T lymphocytes^20^. The importance of HMGB1 in muscle wasting after burn is not understood.

In this study, we aimed to investigate the role of HMGB1 in muscle mass loss after severe burn. For this, a well-established rat model, in which scald burn was induced on 30% TBSA, was used. We examined the effects of burn injury (with and without HMGB1 neutralizing Ab treatment) on muscle loss and evaluated the extent by which protein degradation, autophagy/mitophagy and inflammatory responses constitute pathological mechanisms following severe burn. We discuss potential mechanisms by which HMGB1 neutralizing Ab decreases the systemic effects of burn injury on muscle recovery.

## Materials and methods

### Burn and treatment

Sprague Dawley rats (~ 300 g, 8-weeks old) were purchased from Charles River Laboratories (Wilmington, MA). Animals were housed individually in cages with access to food and water ad libitum*,* and were kept under controlled conditions of temperature, humidity, and 12-h light/dark cycle according to the requirements of the species. All animals were treated humanely.

After at least one-week acclimation, rats were weighed, and randomly distributed to three groups: (1) sham burn (n = 6), (2) burn (n = 8), (3) burn plus treatment (n = 8). The number of animals used in the study were minimum required based on evaluation of the statistical power of sample size using the mean ± standard deviation (SD) values from our previous studies in the burn animal model. These calculations suggested that a sample size of 22 with a minimum of 8 in each of the studied group and 6 in control group would offer 95% confidence interval (CI) to achieve a statistical power of 80% with probability of type 1 error (α) at 0.05 level and observe an effect size (Cohens d) of 1.241. The burn procedure followed the Walker-Mason model with minor modifications^21^. All rats were provided a subcutaneous injection of 0.5 mg/kg buprenorphine 30 min prior to the burn or sham burn procedure. Under anesthesia with 2–4% isoflurane and 5% oxygen inhalation, rats were secured in a prefabricated mold to expose the back and belly, and the exposed shaved skin was immersed in 100 °C water for 10 s on the dorsum and 2 s on the abdomen to produce full-thickness scald cutaneous burn injury. Immediately after burn, animals received resuscitation (15 mL Lactated Ringer’s solution, intraperitoneal) and 0.05 mg/kg buprenorphine for pain management. Further, animals in group 3 and group 2 received one dose of chicken anti-HMGB1 neutralizing polyclonal Ab and isotype control chicken IgY Ab, respectively (2 mg/kg, intraperitoneal, Shino-test, Tokyo, Japan). The selected dosage of the antibody was sufficient to offer immune modulation and phenotypic changes in preliminary studies. Control animals (group 1) underwent the same procedure except that they were immersed in 22–25 °C warm water bath and did not receive fluid resuscitation and analgesia afterwards. The study was conducted in two sets of experiments within 5 months in 2022.

### Measurement of burn surface area

Burn wound surface areas were recorded immediately after injury and at 3-days after burn. Briefly, a piece of transparent polyvinyl wrapping film (Fisher Scientific, Waltham, MA) was placed on the wound area of rat body and a black marker was used to outline the edge of wound for each injured rat. The plastic wrap with the marked wound area and a cm-scale ruler were imaged together by using a Bizhub C3350i printer (Konica Minolta, Wayne, NJ). The digital images were analyzed by ImageJ software (version 1.53, last accessed on November 20, 2022) to record the cm^2^ wound size. The body surface size (cm^2^) was calculated by employing the Meeh-Rubner’s formula kW^(2/3)^, in which k constant = 9.83 and W = rat body weight before injury^22^. The percentage of burn surface area (TBSA) was calculated by [(wound size/body surface size) × 100].

### Tissue harvesting and processing

All animals survived the burn procedure and were euthanized at 3 days after burn injury. Blood samples (~ 7 mL) were directly collected in plastic blood collection tubes with K_2_EDTA (#02-683-99A, Fisher Scientific), and tubes were gently rotated and stored at room temperature. Left hind limb bones (tibia, fibula, and femur) and spleen were stored on ice in 50 mL tubes containing 25 mL ice-cold RPMI-1640 medium (Thermo Fisher, Waltham, MA).

Left hind limb muscles, including lateral gastrocnemius media (GM), gastrocnemius lateral (GL), soleus (SL), and plantaris (PL) on the posterior side and tibialis anterior (TA) and extensor digitorum longus (EDL) on the anterior side, were harvested as described^23^. All muscles were weighed, snap frozen in liquid nitrogen, and stored at − 80 °C for protein and mRNA analysis. A portion of PL and SL muscles from hind left limb were fixed in 10% neutral formalin buffer for histology. Right hind limb muscles were also isolated as above and weighed. A portion of each muscle tissue was desiccated at 55 °C in a DX400 drying oven (Yamato Scientific, Santa Clara, CA) for 3 days, and tissue weight was recorded before and after desiccation.

### Flow cytometry

Blood samples collected in EDTA coated tubes were layered over an equal amount of Histopaque-1077 (Sigma-Aldrich, St. Louis, MO) and centrifuged at room temperature at 400×*g* for 30 min. Peripheral blood mononuclear cells (PBMCs) were obtained from the buffy coat formed at the interface. Spleen tissue sections were macerated by pressing with a plunger of a 3-mL syringe. Splenic cell suspensions were passed through a 70 µm Falcon Cell Strainer (Thermo Fisher) to eliminate clumps and debris and centrifuged at 300×*g* for 4–5 min at 4 °C. Bone marrow (BM) cells were isolated from the femur and tibia by flushing the bone canal with 1X PBS using 5 mL syringe.

Single cell suspensions (PBMC, spleen, and BM cells) were suspended in 1X red blood cell lysis buffer (00-4333-57, eBioscience, San Diego, CA) for 5 min, and lysis was stopped with 30 mL of 1X PBS. Cells were centrifuged at 300×*g* for 5 min and processed for flow cytometry. Briefly, cells (5 × 10^4^ per 100 μL) were washed with flow cytometry staining buffer (00-4222-26, eBioscience) and incubated for 10 min with Fc Blocker (anti-CD16/CD32) in brilliant stain buffer (BD Biosciences, San Jose, CA). Aliquots of cells were incubated in dark with the fluorochrome-conjugated antibodies to surface molecules (concentration determined by titration) for 30 min at 4 °C. Then, cells were washed twice in cold staining buffer, fixed and permeabilized by incubation with fixation and permeabilization solution (BD Biosciences) for 20 min, and washed with perm wash buffer (BD Biosciences)^24^. For analyzing intracellular molecules, cells were stained with monoclonal antibodies against interferon (IFN)-γ, tumor necrosis factor (TNF)-α and IL-1β cytokines for 30 min, washed, and resuspended in staining buffer. All samples were visualized, and data were acquired using the BD LSRII Fortessa flow cytometer. As controls, tubes containing unstained cells and cells incubated with isotype matched IgGs (eBioscience), and FMO (fluorescence minus one) were included. Data were analyzed by using a FlowJo software (v.10.5.3, TreeStar, San Carlo, CA) as described^25^. All antibodies used for flow cytometry are listed in supplemental Table S1.

### Protein extraction and immunoblotting

Approximately 30 mg of frozen gastrocnemius lateral tissue sections from all rats were used. Tissues were homogenized in T-PER lysis buffer (Thermo Fisher), and protein contents were measured by Bradford protein assay (Bio-Rad, Hercules, CA) using a FLUOstar OPTIMA microplate reader (BMG LabTech, Cary, NC). Protein samples (20 µg) were electrophoresed on a 4–15% Mini-Protein TGX gel using a Mini-PROTEAN electrophoresis chamber (Bio-Rad), and proteins were transferred to a PVDF membrane using a Criterion Trans-blot System (Bio-Rad). Membranes were blocked with 5% non-fat dry milk in 50 mM Tris–HCl (pH 7.5)/150 mM NaCl (TBS), washed with TBS-0.1% Tween 20 (TBST) and TBS, and incubated overnight at 4 °C with antigen-specific antibodies diluted to 1:1000 in 5% bovine serum albumin—TBST. Membranes were washed with TBST and TBS, incubated with horseradish peroxidase conjugated secondary antibody (1:10,000 dilution), and images were acquired by using ChemiDoc Touch Imaging System (Bio-Rad). Immunoblots were subjected to Ponceau S staining to confirm equal loading and transferring of samples. Densitometry analysis of protein bands was performed using Image Lab software v6.1.0 (Bio-Rad) and normalized against GAPDH. All antibodies are listed in supplemental Table S2.

### Histology

Hind limb muscle tissue sections (soleus and plantaris) of rats were fixed in 10% buffered formalin, dehydrated in graded ethyl alcohol, cleared in xylene, and embedded in paraffin. Paraffin-embedded 5-micron tissue sections were stained with hematoxylin and eosin (H&E). Slides were imaged at 20X or 60X magnification with an Olympus BX-15 microscope (Center Valley, PA) equipped with digital camera and Simple PCI software (v.6.0, Compix, Sewickley, PA). The inflammatory infiltrate and tissue damage were scored as described previously^26^. Briefly, scoring was defined as (0)—absent/none, (1)—focal or mild with ≤ 1 inflammatory cells foci, (2)—moderate with ≥ 2 inflammatory foci, (3)—extensive with generalized coalescing of inflammatory foci or disseminated inflammation (4)—severe with diffused inflammation, interstitial edema, and loss of tissue integrity^26^. Data were captured from at least four rats per group (two slides per tissue per rat, 36 microscopic fields per slide).

Myofiber size from cross-section plane of the tissue sections was measured by using ImageJ software. Each myofiber was circled in the H&E-stained tissue section under 10X magnification, and the cross-section area of the myofiber was automatically calculated after the image scale was set up with the embedded scale bar. At least 100 myofibers per tissue per rat (n = 4 rats per group) were randomly selected and subjected to myofiber size measurement.

### Statistical analysis

Data were captured in excel and analyzed by using GraphPad Prism v.9.4.0 (San Diego, CA). Shapiro–Wilk or D’Agostino & Pearson Omnibus tests were performed to check the normal distribution of data. The outliers were identified and excluded using ROUT method with Q sets up to 5%. Significance comparing two groups was calculated by Students’ unpaired t test with or without Welch’s correction or non-parametric Mann–Whitney U test. Significance comparing multiple groups was calculated by one-way analysis of variance (ANOVA) with Tukey’s post hoc test or non-parametric Kruskal–Wallis/Dunn’s post hoc test. Data were presented as mean values ± standard error mean (SEM) and significance was accepted at *p* value ≤ 0.05.

### Ethical statement

The National Institutes of Health and ARRIVE guidelines were followed for housing and care of the laboratory animals. All animal experiments were conducted in accordance with the protocols approved by the Institutional Animal Care and Use Committee (protocol number #1811084) at the University of Texas Medical Branch at Galveston.

## Results

### Effects of acute burn injury on body and muscle mass (± HMGB1 Ab)

**Figure 1:**
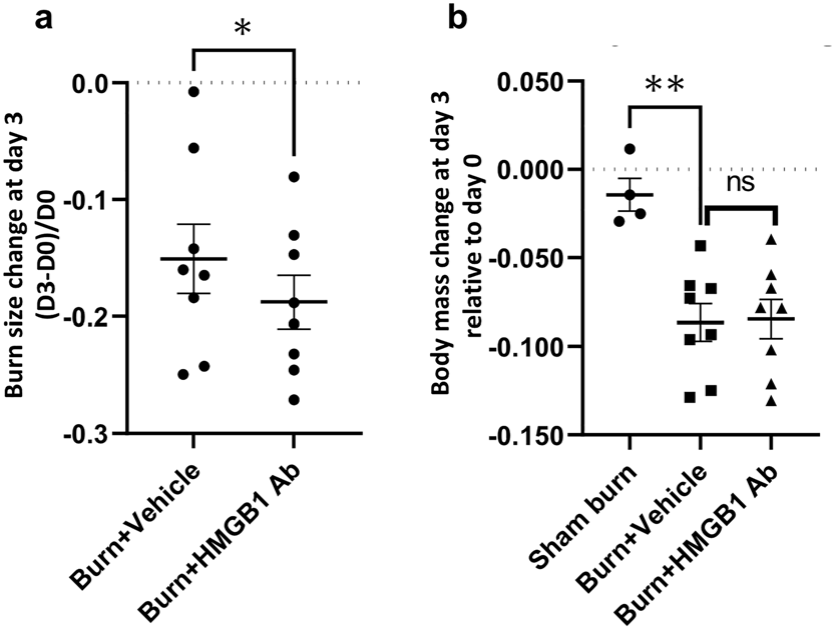
Changes in wound size and body weight post severe burn (± HMGB1 Ab). Sprague Dawley rats were subjected to burn on 30% total body surface area, and immediately after burn injury given one dose treatment with vehicle or anti-HMGB1 antibody. Sham burn animals were included as controls. (**a)** Changes in burn size at day 3 compared to day 0 is shown. **(b)** Percent change in body weight at day 3 relative to day 0 post-burn is shown. Data from each animal are presented as dot plot and mean values ± SEM are shown with horizontal lines (n = 8 rats per group). Significance was calculated by Students’ unpaired t test and *p*-values are annotated as *p < 0.05 and **p < 0.01.

Burn wound on > 30% of TBSA causes significant systemic response including muscle wasting and atrophy in patients^2^. To determine if the animal model used in this study offers reproducible burn wound creation and its effects on the body in presence and absence of HMGB1 Ab treatment, we recorded the wound size, total body mass, and muscle tissue weights for all rats at day 0 and day 3 after burn. Rats exposed to 100 °C water exhibited scald burn on an average of 32.64% ± 0.48% TBSA at day 0. The low SEM values show the consistency of the procedure performance from the two sets of experiments over the 5-month study period. At 3 days after burn injury, burn/treated (vs. burn/non-treated) rats exhibited a significant reduction of wound size (Fig. 1a, 18.77% vs. 15.08%, *p <* 0.05). Total body mass was decreased by 8.65% and 8.46%, respectively, in burn and burn/treated rats, while no change in total body mass was noted in control rats (Fig. 1b).

**Figure 2:**
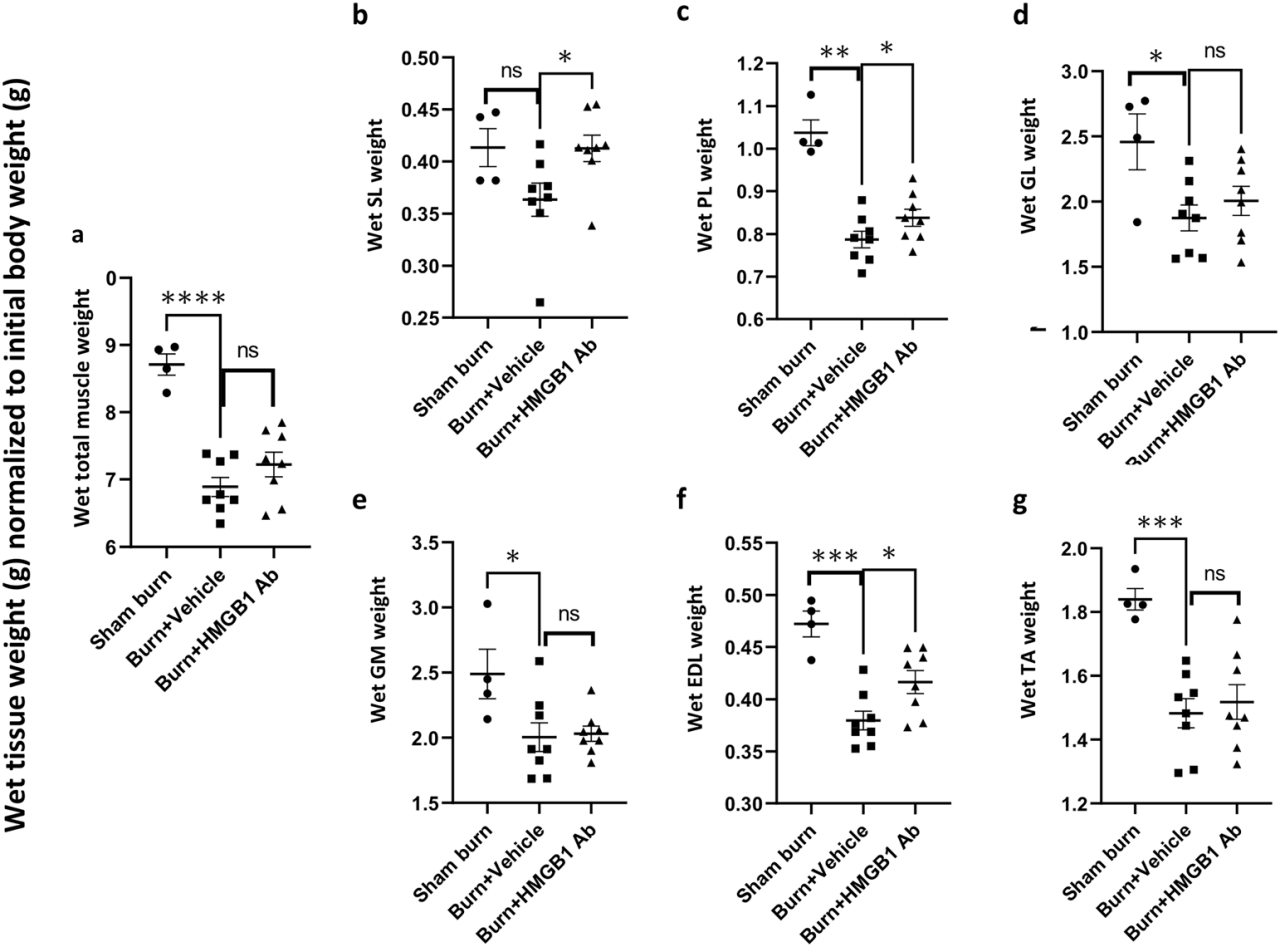
HMGB1 Ab treatment attenuates the burn induced loss in wet weight of the hind limb muscles. Sprague Dawley rats were subjected to burn injury and given one dose treatment with vehicle or anti-HMGB1 antibody (controls: sham burn). Total or individual muscle’s wet weight from both hind limbs at day 3 post-burn, normalized to total body mass at day 0 was calculated. Shown are wet weight of rat’s hind limbs total muscle **(a),** posterior side soleus **(SL, b)**, plantaris **(PL, c)**, gastrocnemius lateral **(GL, d**), lateral gastrocnemius media **(GM, e)** muscles, and anterior side extensor digitorum longus **(EDL, f)** and tibialis anterior **(TA, g)** muscles. Data are presented as dot plot from each rat and mean values ± SEM are shown with horizontal lines (n = 8 rats per group). Significance was calculated by Students’ unpaired t test and *p*-values are plotted as *p < 0.05, **p < 0.01, ***p < 0.001, and ****p < 0.0001.

**Figure 3:**
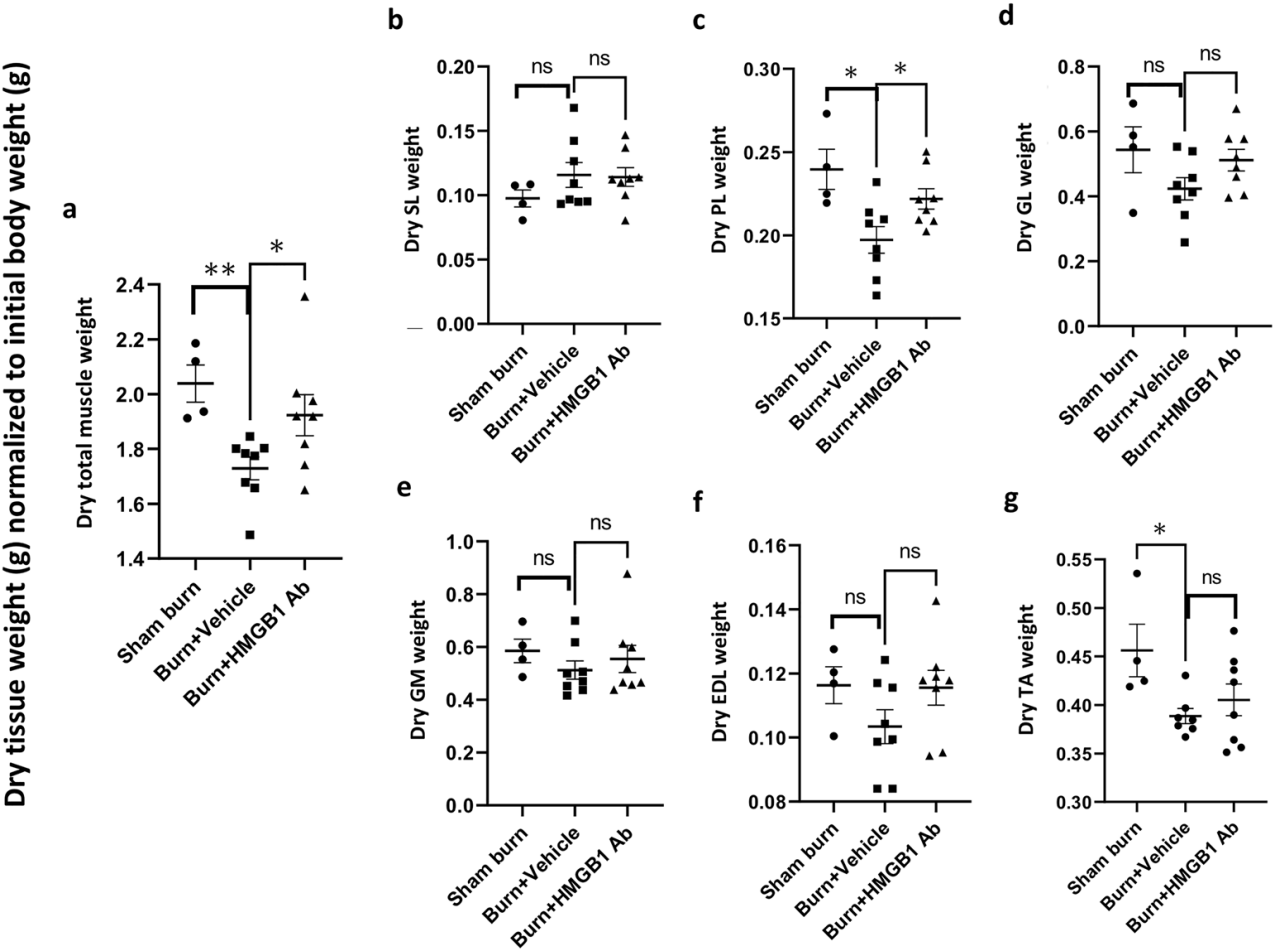
Changes in dry weight of the hind limb muscles post-burn (± HMGB1 Ab). Sprague Dawley rats were subjected to burn on 30% TBSA, and immediately treated with vehicle or anti-HMGB1 antibody (controls: sham burn). Total or individual muscle’s dry weight from both hind limbs at day 3 post-burn, normalized to total body mass at day 0 was calculated. Shown are dry weight of rats’ hind limbs total muscle **(a),** posterior side soleus **(SL, b)**, plantaris **(PL, c)**, gastrocnemius lateral **(GL, d**), and lateral gastrocnemius media **(GM, e)** muscles, and anterior side extensor digitorum longus **(EDL, f)** and tibialis anterior **(TA, g)** muscles. Data are presented as dot plot from each rat and mean values ± SEM are shown with horizontal lines (n = 8 rats per group). Significance was calculated by Students’ unpaired t test and *p*-values are plotted as *p < 0.05 and **p < 0.01.

Next, muscle tissue weight was measured to evaluate the pathophysiological responses of the hind limb muscle following burn injury. Total muscle wet weight as well as the wet weight of individual muscle tissues including soleus (SL), plantaris (PL), gastrocnemius lateral (GL), gastrocnemius media (GM), extensor digitorum longus (EDL) and tibialis anterior (TA) of the hind limbs were significantly decreased by 12.09%–24.14% in burn (vs. sham burn, all, *p <* 0.05) rats at 3 days after injury (Fig. 2a–g). HMGB1 Ab treatment substantially reduced the loss in wet muscle weight evidenced by the finding of preservation of SL muscle weight to normal control level in burn/treated rats (*p <* 0.05), and up to 13.53% improvement in preservation of wet weight of PL, GL, EDL, and TA muscles in burn/treated (vs. burn/non-treated, *p <* 0.05) rats (Fig. 2a–g). Further, HMGB1 Ab therapy was beneficial in preserving the dry weight of the hind limb muscles in burn rats. This was evidenced by the finding that burn on 30% TBSA resulted in 15.19% decline in total dry weight of the hind limb muscles that was reduced to 5.65% only, thus, indicating a 63% recovery of muscle mass in HMGB1 Ab treated (vs. non-treated) burn rats (Fig. 3a). Likewise, burn injury resulted in 17.70%, 22.09%, 12.43%, 11.11%, and 17.46% loss in dry weight of PL, GL, GM, EDL, and TA muscles, respectively, when compared to sham controls (Fig. 3c–g). The burn induced losses in dry weight of the EDL muscle was abolished and that of PL, GL, and GM muscles were significantly decreased in burn/treated rats (5.21%–7.43% vs. 12.43%–22.09%, *p <* 0.05) when compared to burn/non-treated rats (Fig. 3c–g). No effects of burn and HMBG1 treatment were observed on SL muscle dry weight. Myofiber size was decreased by 24.18% in plantaris muscle of burn rats and no other effects on myofiber size of other muscles were observed.

Together, the data presented in Figs. 1, 2 and 3 suggest that the current animal model consistently presents the systemic effects of acute severe burn on muscle mass. Total body mass, total wet and dry weights of the hind limb muscles, as well as wet and dry weights of individual muscles of the hind limb were substantially decreased in burn rats. Treatment with one dose of HMGB1 Ab was effective in reducing the burn size and burn induced losses in wet and dry weights of the hind limb muscles at 3 days after injury. Considering the studied muscles included both fast and slow twitch myofibers and the anatomical position of these muscles support speed, gait, and strength in the hind limb, we surmise that benefits of HMGB1 Ab in averting the loss in muscle mass may preserve muscle function after burn injury.

### Effects of HMGB1 Ab on muscle protein homeostasis in burn rats

**Figure 4:**
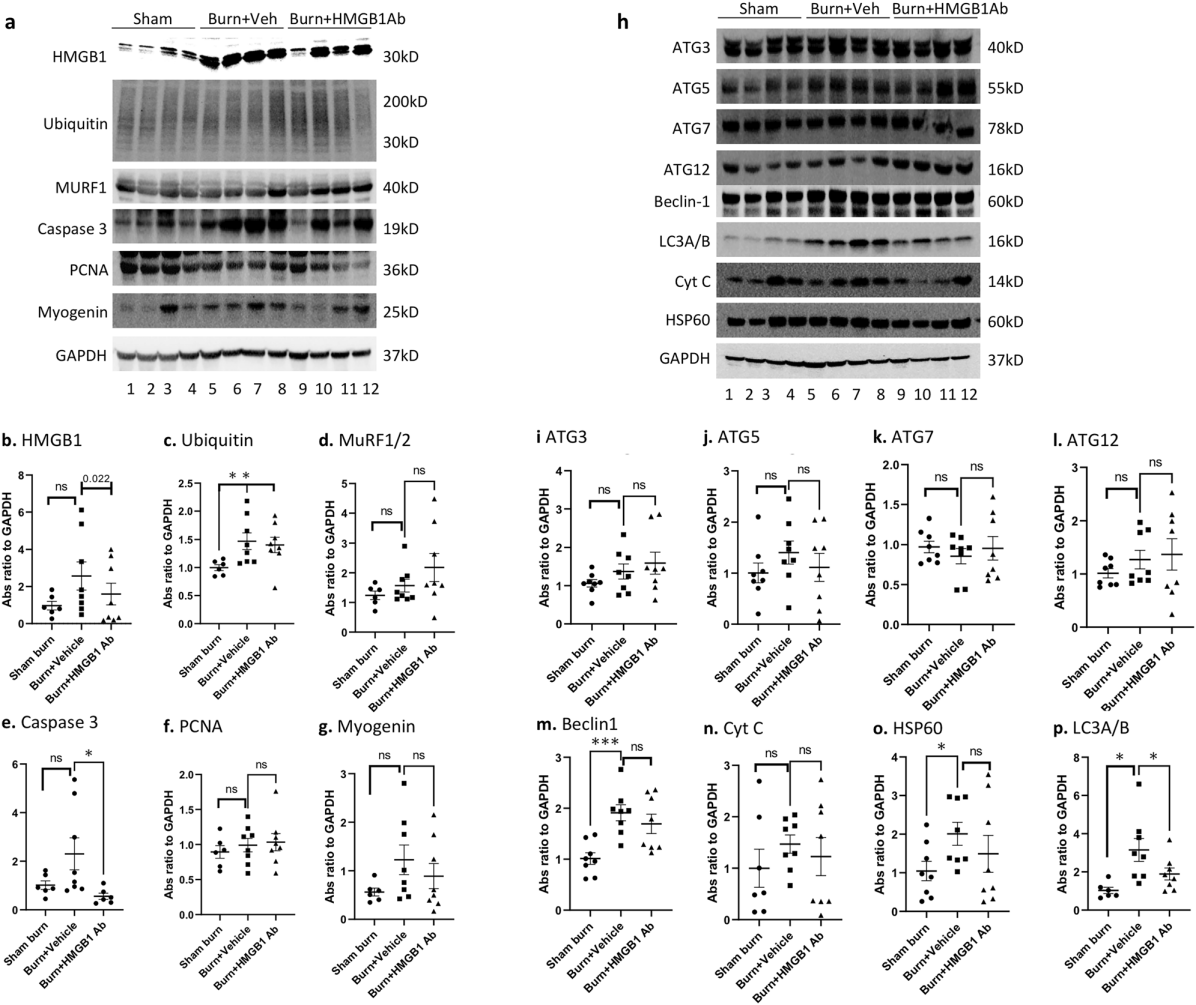
Disturbances of muscle protein homeostasis in burn rats (± HMGB1 Ab). Sprague Dawley rats were subjected to burn, and immediately treated with vehicle or anti-HMGB1 antibody. Hind limb GL muscle homogenates were subjected to Western blotting as described in materials and methods. **(a-g) Protein ubiquitination vs protein synthesis.** Representative Western blot images **(a)** and the relative densitometry score normalized to GAPDH signal are shown for HMGB1 **(b)**, ubiquitin **(c)**, MuRF1/2 **(d)**, cleaved caspase 3 **(e)**, PCNA **(f)**, and myogenin **(g)** proteins. **(h–p) Autophagy signaling.** Representative Western blot images **(h)** and the relative densitometry score normalize to GAPDH signal are shown for ATG3 **(i)**, ATG5 **(j)**, ATG7 **(k)**, ATG12 **(l)**, Beclin1 **(m)**, cytochrome c **(n)**, HSP60 **(o)** and LC3A/B **(g)** proteins. Data in b-g and i-p are presented as dot plot from each rat and mean values ± SEM are shown with horizontal lines (n = 6 rats in control group and 8 rats each in burn and burn/treated groups). Significance was calculated by Students’ unpaired t test with or without Welch’s correction or Mann–Whitney U test and *p*-values are plotted as *p < 0.05, ** *p <* 0.01, and *** *p <* 0.001.

Imbalance in the protein degradation and protein synthesis machinery constitutes a major mechanism of muscle cachexia after burn injury^12^. To understand the regulatory mechanisms of muscle homeostasis with HMGB1 signal modulation, we first examined the expression levels of proteins involved in cell death and myogenesis in gastrocnemius lateral muscle of sham burn, burn, and burn/HMGB1 Ab treated rats by western blotting. Representative images are presented in Fig. 4a, and densitometry analyses of protein bands of interest (normalized to GAPDH) are shown in Fig. 4b–g. We noted 164.80% and 64.87% increase in HMGB1 levels in burn and burn/treated rats, respectively (vs. sham controls), thus, indicating that one dose of HMGB1 Ab reduced the HMGB1 levels by 60.6% in burn/treated (vs. burn/non-treated, *p <* 0.05) rats at 3 days after burn (Fig. 4b). E3 ubiquitin protein ligases (also named Muscle Ring-Finger protein (MuRF1/2)) along with protein cleaving caspase 3 enhance the ubiquitin mediated protein degradation^27^. Other proteins, e.g., proliferating cell nuclear antigen (PCNA) and myogenin play a direct role in skeletal muscle development and repair^28^. The extent of protein ubiquitination (1.47 ± 0.15 vs. 1.00 ± 0.05, 47.06% increase, *p <* 0.05), and MuRF1/2 (1.57 ± 0.21 vs. 1.24 ± 0.14) and caspase 3 (2.30 ± 0.66 vs. 1.02 ± 0.18, 126.43% increase, *p <* 0.05) levels were substantially increased in burn vs. sham burn rats, while muscle levels of PCNA and myogenin were not altered or slightly increased in burn (vs. sham control) rats (Fig. 4c–g). Treatment with HMGB1 Ab did not alter the extent of protein ubiquitination and MuRF1/2, PCNA, or myogenin protein levels in the GL muscle of burn rats. Yet, burn induced increased expression of active caspase 3 that controls the cell death program was completely abolished after HMGB1 Ab treatment of burn rats (2.30 ± 0.66 vs. 0.56 ± 0.13, burn vs. burn/treated, *p <* 0.05) (Fig. 4e).

Autophagy is a quality control process involved in removal of unnecessary or dysfunctional proteins and organelles in autophagosomes through a lysosome-dependent mechanism^29^. We, therefore, monitored whether HMGB1 signals autophagy pathways to alter muscle mass loss after burn. Representative images of autophagy protein levels are presented in Fig. 4h**,** and densitometry analyses of protein bands of interest (normalized to GAPDH) are shown in Fig. 4i–p. Except for ATG7 that was not altered after burn, other autophagy related proteins (ATG3, ATG5, ATG12) exhibited a moderate increase in expression in burn rats, however, this was not significant when compared to sham burn rats, and these proteins did not change in expression after HMGB1 Ab treatment (Fig. 4i–l). Yet, expression of Beclin 1 that signals autophagy and mitochondrial stress proteins including cytochrome c and heat shock protein 60 (HSP60) that signal cell death response were increased in burn (vs. sham control) rats by 88.66% (*p <* 0.001), 49.13% (*p <* 0.05), and 92.09% (*p <* 0.05), respectively (Fig. 4 m–o). Consequently, endogenous level of LC3A/B (catalyzes protein degradation) was also increased by 206% in GL muscle of burn rats when compared to sham burn rats (3.15 ± 0.60 vs. 1.03 ± 0.17, *p <* 0.05, Fig. 4p). Treatment with HMGB1 Ab resulted in substantially lower levels of burn-induced increases in Beclin 1, cytochrome c, HSP60, and LC3A/B proteins than was observed in burn rats (24.43%–84.10% vs. 49.13%–206.85%). Together, the results presented in Fig. 4 suggest that acute severe burn induced mitochondrial stress (cytochrome c, HSP60), cell death (caspase 3) and protein ubiquitination/autophagy (LC3A/B) that together contribute to protein degradation in muscle tissues. Treatment with anti-HMGB1 Ab, at least partially, reduced the burn induced muscle proteolysis by modulating the cell death and autophagy in acutely burn rats.

### Effects of HMGB1 neutralizing antibody on tissue inflammatory infiltrate in burn rats

**Figure 5:**
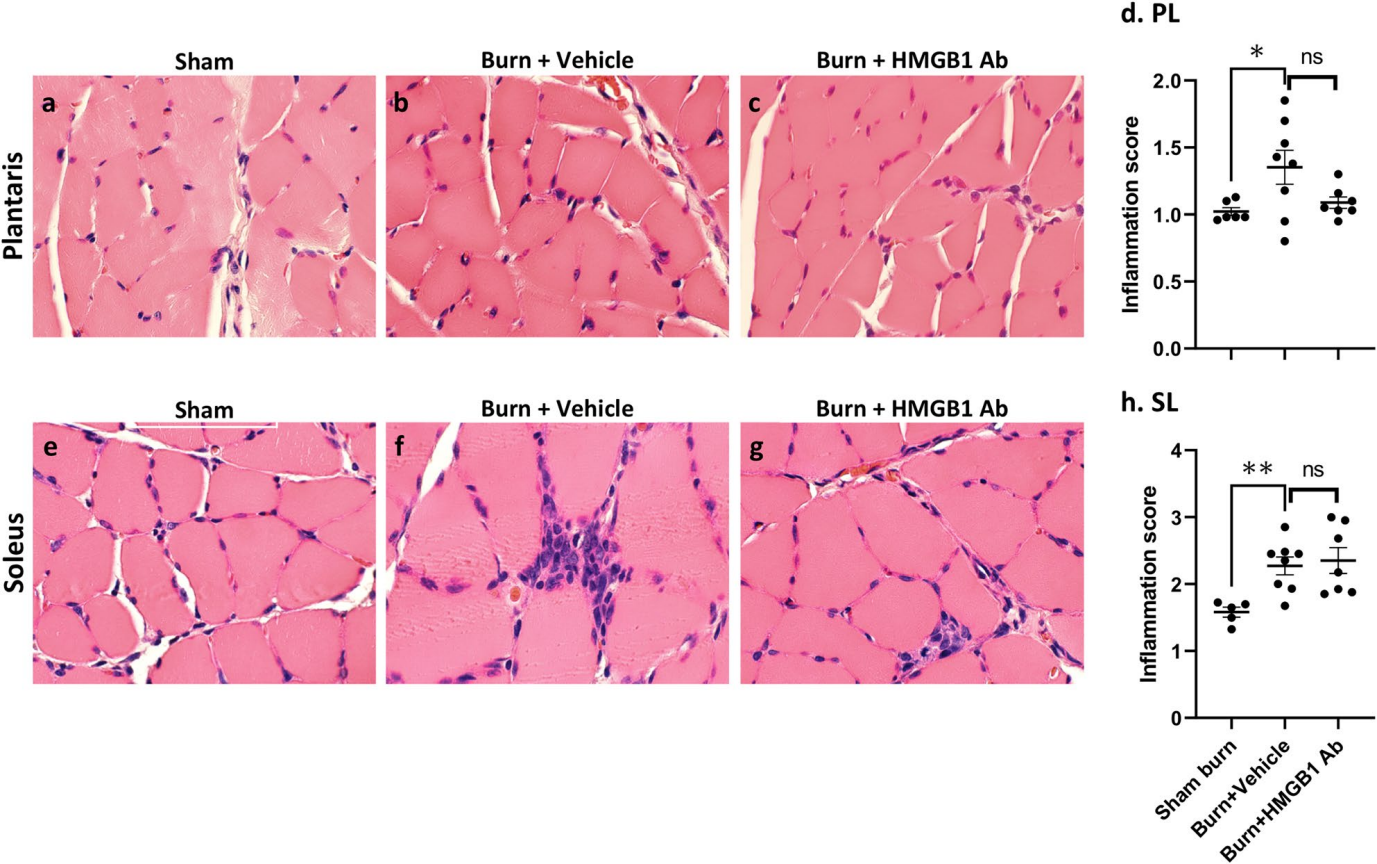
Histological evaluation of inflammatory infiltrate in hind limb muscles of burn rats (± HMGB1 Ab). Sprague Dawley rats were subjected to burn, and immediately treated with vehicle or anti-HMGB1 antibody (controls: sham burn). Paraffin-embedded hind limb tissue Sects. (5 μM) were examined by hematoxylin/eosin staining (blue: nuclear; pink: muscle/cytoplasm). Shown are representative H&E-stained images of plantaris **(a–c)** and soleus **(e–g)** muscle tissue sections of sham/no burn** (a, e)**, burn/vehicle **(b, f)**, and burn/HMBGB1 Ab **(c, g)** rats. Average histological score values of plantaris **(d)** and soleus **(h)** muscles were derived from analysis of n = 4 rats per group with two slides per tissue per rat, 36 microscopic fields per slide per experiment. Data in d & h are presented as dot plot and mean values ± SEM are shown with horizontal lines. Significance was calculated by one way ANOVA with Tukey’s post-hoc test and *p*-values are plotted as *p < 0.05 and **p < 0.01.

Secreted HMGB1 has been shown to promote inflammation in infection^30^. Burn trauma increased the HMGB1 release in patients^18^ and rats (Fig. 4b). We, therefore, determined if treatment with HMGB1 Ab altered the inflammatory stress in muscle tissues of burn rats. Histological evaluation of muscle tissue sections showed ~ 30% increase in the inflammatory infiltrate in plantaris muscle of burn rats as compared to that noted in sham burn rats (Fig. 5a–d, score: 1.31 ± 0.08 vs. 1.01 ± 0.05, *p <* 0.5). Likewise, inflammatory infiltrate in soleus muscle of burn rats was increased by 42.7% as compared to the sham burn rats (Fig. 5**e–h**, score: 2.27 ± 0.08 vs. 1.59 ± 0.07, *p <* 0.01). Treatment of burn rats with HMGB1 Ab had no significant effect on soleus muscle levels of inflammatory infiltrate (score: 2.27 ± 0.08 vs. 2.35 ± 0.11, burn vs burn/treated, Fig. 5e–h); however, HMGB1 Ab substantially decreased the inflammatory infiltrate in plantaris muscle tissue of burn rats (score: 1.31 ± 0.08 vs. 1.11 ± 0.05, burn vs burn/treated, Fig. 5a–d). These data suggest that HMGB1 promotes the inflammatory infiltrate in muscle tissues of acutely burn rats, and this can be diminished by HMGB1 neutralizing Ab.

### Myeloid cells and T cells response in burn rats (± HMGB1 Ab)

**Figure 6:**
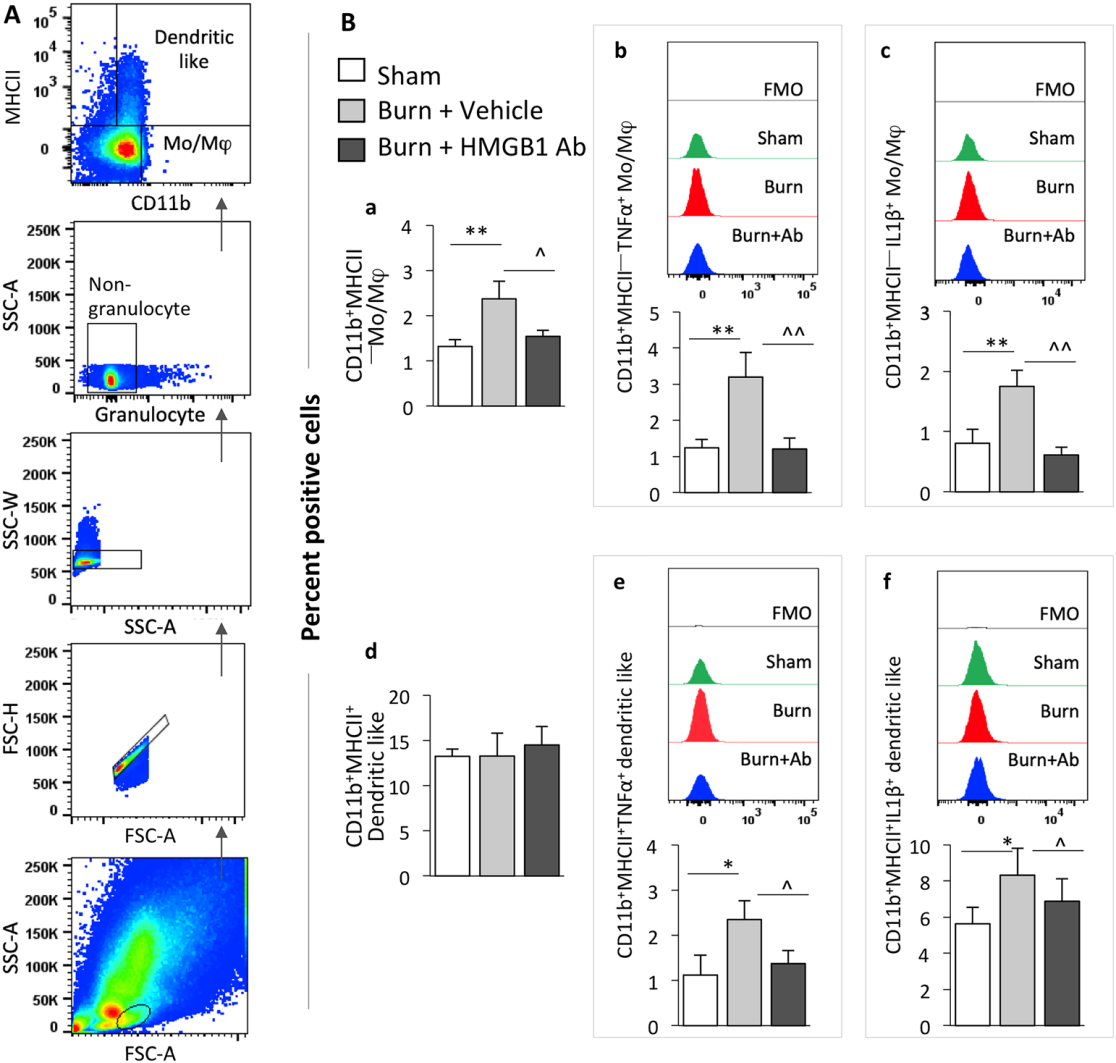
Effect of burn injury on innate immune cell response in burn rats (± HMGB1 Ab). Sprague Dawley rats were subjected to burn, immediately treated with one dose of vehicle or anti-HMGB1 antibody and euthanized at 3 days post-burn (controls: sham burn). Single cell suspensions of bone marrow cells were labeled with fluorescent-conjugated antibodies and analyzed by flow cytometry. **(A)** Representative gating strategy is shown. BM cells of interest were gated in forward (FSC-A) and side (SSC-A) scatter to capture the single cells and exclude the doublets or clumps. Single cells were further gated to exclude the granulocytes and capture non-granulocytes. Non-granulocytes were gated for CD11b and MHCII to capture the CD11b^+^MHCII^-^ monocyte/macrophage (Mo/Mφ) and CD11b^+^MHCII^+^ dendritic like myeloid cell populations. **(B)** Percent positive CD11b^+^ myeloid cells that were MHCII^-^ (**a**) or MHCII^+^ (**b**) and expressed TNFα (**b, e**) or IL-1β (**c, f**) intracellular cytokines are shown. All data are derived from *n* ≥ 6 rats per group. Significance was calculated by 1-way ANOVA/Tukey’s post-hoc test or Kruskal–Wallis/Dunn’s post-hoc test and annotated as * (sham vs. burn or burn/treated) and ^ (burn vs. burn/treated). One and two symbol characters were used to represent the *p*-values of ≤ 0.05 and ≤ 0.01, respectively.

Severe burn patients have an extended inflammatory response with elevated serum levels of IFNγ and TNFα^31^. However, it is not clear as to which types of immune cells contribute to the inflammatory state after burn injury. We, therefore, enumerated the immune cells profile to examine how acute burn triggers the inflammation and if HMGB1 Ab treatment modulates the innate immune cells or T cells response after injury. Detailed results including mean ± SEM values from immune profiling of bone marrow (BM) myeloid cells, PBMCs and splenocytes are shown in supplementary Table S3. Gating strategy for flow cytometry analysis of BM myeloid cells is shown in Fig. 6A. These data showed that BM myeloid cells’ differentiation to committed monocytes (Mo: CD11b + MHCII-) as well as their functional activation, i.e., inflammatory cytokines (TNFα, IL1β) production, was increased by 79%, 158% and 116%, respectively, in burn rats as compared to that noted in sham controls (Fig. 6A a–c, all, *p <* 0.01). Likewise, the frequencies of TNFα- and IL1β- expressing CD11b^+^MHCII^+^ dendritic like myeloid cells were increased by 109% and 47.7%, respectively, in burn (vs. sham burn) rats (Fig. 6B d–f). HMGB1 Ab treatment completely abrogated the burn-induced increases in the frequencies of the monocytes/macrophages (Mo/Mφ), TNFα- and IL6- producing Mo/Mφ, as well as TNFα producing dendritic like myeloid cells and significantly decreased the frequency of IL6 expressing dendritic like myeloid cells in burn/treated (vs. burn/non-treated, all, *p <* 0.05) rats (Fig. 6B a–f).

**Figure 7:**
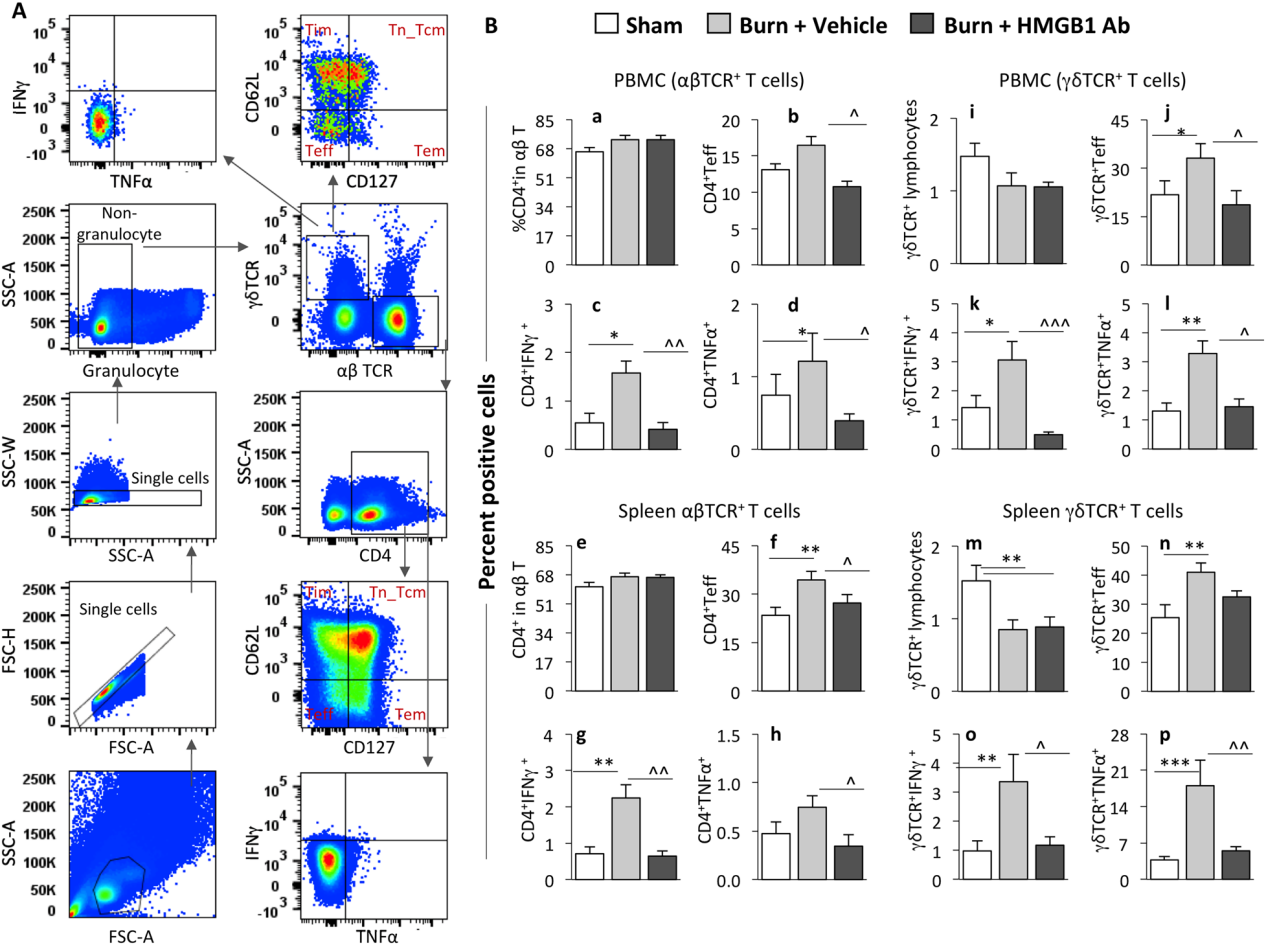
Effect of burn injury on T cell response in burn rats (± HMGB1 Ab). Sprague Dawley rats were subjected to burn, immediately treated with one dose of vehicle or anti-HMGB1 antibody and euthanized at 3 days post-burn (controls: sham burn). Single cell suspensions of peripheral blood mononuclear cells (PBMC) and splenocytes were labeled with fluorescent-conjugated antibodies and analyzed by flow cytometry. **(A)** Representative gating strategy is shown. The cells of interest were gated in forward (FSC-A) and side (SSC-A) scatter to exclude the doublets and cell clumps and capture the single cell population. Single cells were then gated to exclude granulocytes and capture non-granulocyte cells. Non-granulocytes were gated for αβ TCR^+^ and γδ TCR^+^ T cell sub-populations. The αβ T cells were further gated to capture CD4^+^ T cells. Both αβ CD4^+^ T cells and γδ T cells were subjected to CD127 and CD62L gating for the identification of activation phenotypes (effectors (Teff): CD62L^-^CD127^-^, effector memory (Tem): CD62L^-^CD127^+^, intermediate (Tim): CD62L^+^CD127^-^, and naïve/central memory (Tn_Tcm): CD62L^+^CD127^+^) and intracellular cytokines TNFα and IFNγ expression. **(B)** Percentages of αβ CD4^+^ T cells **(a–b, e–f)** and γδ T cells **(i-j, m–n)** that exhibited effector phenotype and intracellular IFNγ and TNFα production in PBMCs **(c-d, k-l)** and spleen **(g-h, o-p)** of sham, burn/non-treated, and burn/HMGB1 Ab-treated rats are shown. Data in bar graphs are derived from n ≥ 6 rats per group and plotted as mean values ± SEM. Significance was calculated by 1-way ANOVA/Tukey’s post-hoc test or Kruskal–Wallis H/Dunn’s post-hoc test and annotated as * (sham vs. burn or burn/treated) and ^ (burn vs. burn/treated). One, two, and three symbol characters were used to present the *p*-values of ≤ 0.05, ≤ 0.01, and ≤ 0.001, respectively.

The αβ T cell receptor (TCR) positive T cells are generally major histocompatibility complex (MHC) restricted and respond to antigenic stimulation and γδ TCR positive T cells are MHC independent and can rapidly respond to injurious stimuli. Some studies showed an increase in αβ T and γδ T cells in the circulation of patients with severe systemic inflammatory response syndrome^32^; however, little is known regarding the role of T cell subsets in burn injury. Gating strategy for flow cytometry analysis of PBMCs and splenocytes for the characterization of αβ T and γδ T cell subsets is shown in Fig. 7A. The CD4^+^ T cells constituted up to 70% of the circulatory and splenic αβ T lymphocytes in rats, and CD4^+^ T cells frequencies were not changed in burn and burn/HMGB1 Ab treated rats (Fig. 7B a and e). Yet, the percentages of αβ CD4^+^ T cells that acquired the CD62L^-^CD127^-^ effector (Teff) phenotype and produced inflammatory cytokines (IFNγ >  > TNFα) were increased by 25.6% and 61.3%–187% (*p <* 0.05), respectively, in circulation (Fig. 7B b–d) and 46.7% (*p <* 0.01) and 59.6%–215% (*p <* 0.01), respectively, in spleen (Fig. 7B f–h) of burn (vs. sham) rats. The burn induced increase in αβ CD4^+^ T cells’ differentiation to effector phenotype and proinflammatory cytokines expression was abrogated when burn rats were treated with single dose of HMGB1 Ab (Fig. 7B b–d and f–h, all, *p <* 0.05).

The γδ T cells constituted < 2% of the total lymphocytes in rats, and the overall frequency of γδ T cells was decreased by 27.7%–41.4% in circulation and spleen of burn and burn/treated rats (Fig. 7B i and m). Despite an overall decline, the γδ T cells differentiation to CD62L^-^CD127^-^ Teff phenotype and functional activation (TNFα >  > IFN-γ) were increased by 52.1% (*p <* 0.05) and 115%–150% (*p <* 0.05), respectively, in circulation (Fig. 7B j–l) and 30.6% (*p <* 0.01) and 243%–383% (*p <* 0.01), respectively, in spleen (Fig. 7B n–p) of burn rats. Again, HMGB1 Ab treatment abolished the burn induced increases in the frequencies of γδ Teff cells, and proinflammatory cytokines producing γδ Teff cells in burn rats (Fig. 7B k–l and o–p, *p* < 0.05–0.01).

**Figure 8:**
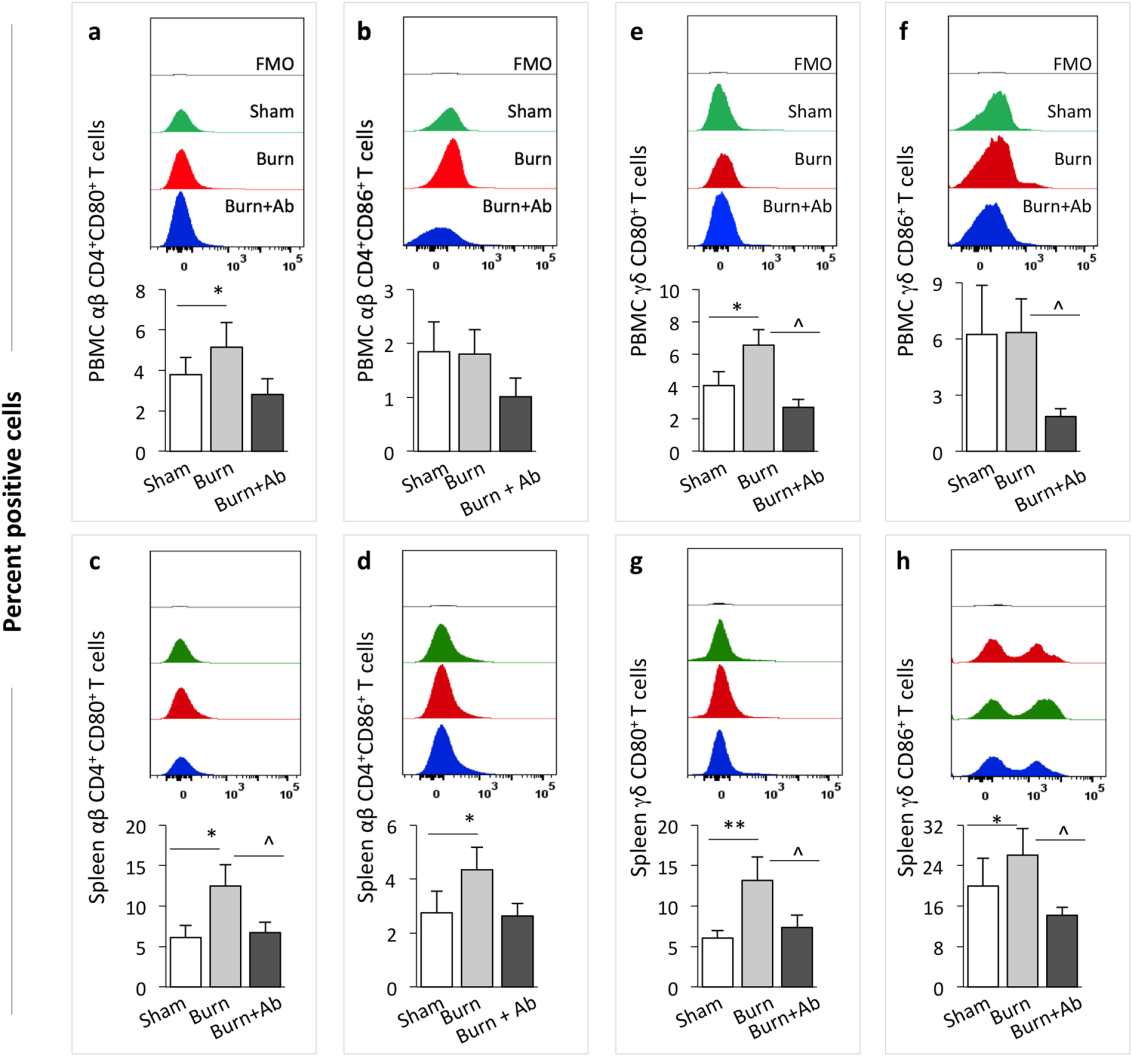
T cells expression of costimulatory molecules in burn rats (± HMGB1 Ab). Sprague Dawley rats were subjected to burn, immediately treated with one dose of vehicle or anti-HMGB1 antibody and euthanized at 3 days post-burn (controls: sham burn). Single cell suspensions of peripheral blood mononuclear cells (PBMC) and splenocytes were labeled with fluorescent-conjugated antibodies and analyzed by flow cytometry. Both αβ CD4^+^ T cells and γδ T cells were analyzed for the expression of CD80 and CD86 co-stimulation markers. Percentages of T lymphocytes that were αβ CD4^+^CD80^+^ (**a, c**), αβ CD4^+^CD86^+^ (**b, d**), γδ CD80^+^ (**e, g**) and γδ CD86^+^ (**f, h**) in peripheral blood (**a, b, e, f**) and spleen (**c, d, g, h**) of sham, burn, and burn/HMGB1 Ab-treated rats are shown. Representative images above the bar graphs show the mean fluorescence intensities of the target surface molecules in each group of rats. Data in bar graphs are derived from n ≥ 6 rats per group and plotted as mean values ± SEM. Significance was calculated by 1-way ANOVA/Tukey’s post-hoc test or Kruskal–Wallis H/Dunn’s post-hoc test and annotated as * (sham vs. burn or burn/treated) and ^ (burn vs. burn/treated). One, two, and three symbol characters were used to represent the *p*-values of ≤ 0.05, ≤ 0.01, and ≤ 0.001, respectively.

The CD80 and CD86 costimulatory glycoproteins are known to be expressed on antigen presenting cells (APC). Recently, CD80 and CD86 were found to be expressed on T cells in APC-free conditions^33^; yet little progress has been made in understanding their significance, especially in acute burn injury. Flow cytometry analysis showed that percentages of CD80 and CD86 expressing αβ CD4^+^ T cells were increased by 35.6%–104.2% in peripheral blood or spleen of burn rats (Fig. 8a–d, *p <* 0.05). Likewise, CD80 and CD86 expressing γδ T cells were increased by 30.02%–118.24% in peripheral blood or spleen of burn rats (Fig. 8e–h, *p <* 0.05–0.01). Treatment with HMGB1 Ab eliminated the burn induced increase in the CD80 and CD86 expression on both αβ and γ∂ T cell subsets in the peripheral blood and spleen of the burn rats (Fig. 8a–h, all *p <* 0.05).

Together, the results presented in Figs. 6, 7, and 8 suggest that HMGB1 plays an important role in differentiation, maturation and proinflammatory activation of myeloid cells in the bone marrow and αβ and γδ T lymphocytes in peripheral and lymphatic immune systems after the acute burn injury; and neutralization of HMGB1 would have potential beneficial effects in controlling the immune cells activation and dysregulated inflammation in burn trauma.

## Discussion

In this study, we observed that acute burn on 30% total body surface area caused muscle mass loss in rats. Local inflammation and protein degradation responses in muscle tissues along with the systemic activation of proinflammatory myeloid cells and T cells were also observed in burn rats at 3 days after burn. Neutralizing the soluble HMGB1 levels by treatment with a single dose of antigen-specific Ab alleviated the acute muscle mass loss in burn rats. HMGB1 Ab did not facilitate the muscle cells proliferation and myogenic differentiation and did not prevent the ubiquitin-proteolysis in muscle tissues of burn rats. Instead benefits of the HMGB1 Ab treatment in preserving the muscle mass were delivered, at least in part, by controlling the systemic activation of proinflammatory immune cells, decreasing the muscle infiltration of inflammatory infiltrate, and preventing the autophagy and cell death responses.

Under physiological conditions HMGB1 maintains the DNA replication and repair in the nucleus^15–17^. HMGB1 released from activated or apoptotic immune and non-immune cells can act as an endogenous danger molecule and signal innate immune system by interacting with pattern recognition receptors^34^. Because of its function as an extracellular alarmin, HMGB1 has emerged as a clinical biomarker for several disorders^16,35^. HMGB1 release was markedly increased within 6 h post injury in trauma patients with ISS ≥ 15^36^. Plasma HMGB1 levels were increased in patients with burn on > 10% TBSA at the time of hospitalization, and the secreted HMGB1 levels correlated with burn size and poor survival outcomes in burn patients^18^. HMGB1 level in gastrocnemius lateral muscle of rats exposed to burn on 30% TBSA (vs. sham burn) was increased by 164.80%, similar to what was observed in our previous study of burn mouse model^37^ and treatment with HMGB1 Ab improved the muscle mass in burn rats. These findings provide evidence for the pathological role of burn induced systemic increase in HMGB1 in muscle loss and imply that HMGB1 neutralization will prevent the muscle mass loss and muscle wasting after burn. Other studies testing the neutralizing antibody treatment at different time-points postburn will be needed to substantiate the pathological significance of HMGB1 in muscle loss after burn.

Disruption of muscle metabolic and cellular homeostasis is believed to be the main cause of muscle wasting and atrophy in cancer^38^, and it was also noted in burn rats in this study. Our observations in published reports^9,10,39^ and this study showed that cell death, protein ubiquitination and autophagy pathways associated with proteolysis were partially activated, and not compensated by an increase in the muscle protein synthesis after burn. Increase in mitochondrial fragmentation associated cell death was also noted in C2C12 myoblasts incubated with serum of burn rodents^10^. Treatment with HMGB1 Ab modulated the expression of markers of autophagy and cell death but it had insignificant effects on burn induced increase in protein ubiquitination or in activating the cell growth/myogenesis at day 3 after burn. HMGB1/autophagy mediated muscle atrophy was also documented in de-nervated mice^40^. Hyperactivation of autophagy has also been noted to contribute to muscle loss in several critical conditions including cancer cachexia, sepsis, cirrhosis, and chronic obstructive pulmonary disease^41^. Further studies will be needed to investigate if HMGB1 translocation to muscle tissues directly influenced the muscle protein homeostasis or if HMGB1 induction of local inflammation and consequent disturbances of mitochondrial biogenesis and cell survival (vs. cell death) pathways^42^ contributed to muscle loss after burn. Likewise, additional studies will be needed to evaluate the long-term effects of HMGB1 in modulating the muscle biogenic responses after burn.

Studies have reported that HMGB1 regulates muscle fate through engaging the receptors for advanced glycation end products (RAGE) and toll-like receptors (TLRs), though the downstream signaling mechanisms involved in modulating the muscle homeostasis are note entirely understood. Mori et al.^43^ reported HMGB1 accumulated in skeletal muscle after hind limb ischemia in mice, wherein it signaled skeletal muscle regeneration and neovascularization in a RAGE-dependent manner. Using RAGE deficient (vs. competent) C2C12 myoblasts, Riuzzi et al.^44^ showed that HMGB1-RAGE-signaling enhanced the myogenin expression that, in turn, suppressed the PAX7 (Paired Box 7) transcription factor, and thereby accelerated the muscle regeneration while limiting the muscle cells proliferation and self-renewal. Conversely, elevated serum levels of HMGB1 were noted in cachexia than non-cachexia colon cancer patients, and exosomes generated by the CT26 colon cancer cells induced muscle wasting like phenotype in C2C12 myotubes and mice via the activation of TLR4-nuclear factor kappa B (NF-κB) pathway^45^. Consequently, muscle wasting in CT26 mouse model was relieved by glycyrrhizin (inhibits HMGB1) treatment^45^. Likewise, HMGB1 signaled TLR4/NF-κβ pathway in alcohol induced skeletal muscle atrophy in zebrafish^46^. We observed no effects of HMGB1 in controlling the myogenesis in muscle tissue of burn mice. Future studies investigating the spatial distribution of native and post-translationally modified isoforms of HMGB1 and their specific role in regulating RAGE, TLRs, and other receptors will be needed to delineate the signaling mechanisms by which HMGB1 influences the muscle regeneration vs atrophy after burn injury.

Local and systemic inflammation are the major pathological events post burn injury^2,47^. Valvis et al.^48^ noted a rapid increase in the cytokines, monocytes, and neutrophils in the peripheral blood and activated CD4^+^ and CD8^+^ T cells in the inguinal draining lymph nodes (ILNs) of mice at 3 days post-burn; and found that the peripheral CD8^+^T cells persisted up to 84 days post-burn. Moins-Teisserenc et al.^49^ conducted longitudinal analysis of severely ill adult burn patients over a period of 28 days post-admission (vs. healthy donors) and identified increased frequencies of Human Leukocyte Antigen – DR isotype positive monocytes associated with bacterial infection and septic shock, upregulation of adaptive T cells (CD4^+^ and CD8^+^) and a decline in the frequencies of unconventional lymphocytes such as γδ T cells and invariant natural killer T cells in critically burn patients at the time of admission. Further, continuance of altered immune status or absence of immune recovery patterns were associated with poor prognosis. Rats with burn on 30% TBSA in this study exhibited the characteristic features of immune activation profile as has been noted in burn patients. We found that the circulatory and splenic frequencies of γδ T cells were significantly decreased in burn rats, while the frequency of bone marrow myeloid cells and peripheral and splenic frequencies of proinflammatory CD4^+^T cells were significantly increased in response to burn. Local immune cell infiltration was also increased in rat muscle tissue at 3 days after burn. The immune cells’ expression of proinflammatory cytokines such as IFNγ and TNFα that can signal muscle degradation and muscle cell death^9^ or impair myogenesis and protein synthesis^50^ was also noted in burn rats. These findings suggest that proinflammatory state may directly (or in synergy with autophagy-mediated protein degradation) dysregulate the muscle homeostasis and contribute to muscle loss after burn injury.

Besides regulating the muscle regeneration (vs. degeneration), HMGB1-RAGE are shown to signal maturation and migration of dendritic cells and activation of T cells, and thereby function as a significant mediator of sterile inflammation^51^. A recent study showed that treated with non-oxidizable form of HMGB1 reduced the inflammation and fibrosis and promoted muscle function in mouse model of muscular dystrophy^52^. In this study, HMGB1 role in regulating the immune cell activities was shown by the findings that treatment with HMGB1 neutralizing Ab nullified the proinflammatory activation of bone marrow myeloid cells and the αβ CD4^+^ T and γδ T lymphocytes in periphery and lymphoid organ of burn mice. Whether HMGB1 neutralization prevented the TLR/RAGE signaling of immune activation in acute burn, and whether HMGB1 Ab therapy will offer long-term benefits in establishing immune and muscle homeostasis post-burn in muscle-use and -disuse conditions remain to be investigated in future studies.

## Conclusions

The current study delineated the effect of HMGB1 in dysregulating the immune response and muscle integrity following burn injury. The presented results confirm the association between cachectic muscle wasting and activated innate and adaptive immunity after severe burn. Further, it was found that neutralization of HMGB1 improved the immune recovery and muscle mass by reducing the proinflammatory cytokines (IFNγ, IL1β, and TNFα) expression in myeloid cells and αβ CD4^+^ T, and γδ T lymphocytes and by inhibiting the autophagy-cell death pathway of protein degradation. The current study suggest the ant-HMGB1 therapies may have therapeutic potential in preventing the muscle loss after burn injury.

One limitation of this study is that beneficial effects of HMGB1 Ab were monitored at only one time-point after burn. Further studies are needed to establish if HMGB1 Ab treatment (single vs. multiple doses, various amounts, time of treatment) would be beneficial in regulating the longitudinal immune profile and muscle loss following burn injury. We observed significant wound size reduction in burn/ HMGB1 Ab treated (vs. burn/non-treated) rats, which implies that the HMGB1 neutralization after burn might also be beneficial in enhancing the wound closure. This observation, if validated in future studies, would indicate the future development of topical anti-HMGB1 treatments to improve wound closure.

## Supplementary Information

Supplementary Legends.

Supplementary Figure S1.

Supplementary Figure S2.

Supplementary Table S1.

Supplementary Table S2.

Supplementary Table S3.

## Data Availability

All relevant data are available within the presented manuscript. Any material and information generated during the study will be available for sharing with other researchers under appropriate institutional agreements. Any inquiries should be directed to corresponding authors Drs. Song and Garg.

## References

[1] Peck MD (2011). Epidemiology of burns throughout the world. Part I: Distribution and risk factors. Burns.

[2] Jeschke MG (2008). Pathophysiologic response to severe burn injury. Ann. Surg..

[3] Hart DW (2000). Persistence of muscle catabolism after severe burn. Surgery.

[4] Newsome TW, Mason AD, Pruitt BA (1973). Weight loss following thermal injury. Ann. Surg..

[5] Chang DW, DeSanti L, Demling RH (1998). Anticatabolic and anabolic strategies in critical illness: A review of current treatment modalities. Shock.

[6] Hart DW (2000). Determinants of skeletal muscle catabolism after severe burn. Ann. Surg..

[7] Padfield KE (2006). Local and distant burn injury alter immuno-inflammatory gene expression in skeletal muscle. J. Trauma.

[8] Merritt EK, Cross JM, Bamman MM (2012). Inflammatory and protein metabolism signaling responses in human skeletal muscle after burn injury. J. Burn. Care Res..

[9] Song J, Saeman MR, De Libero J, Wolf SE (2015). Skeletal muscle loss is associated with TNF mediated insufficient skeletal myogenic activation after burn. Shock.

[10] Sehat A (2017). Burn serum stimulates myoblast cell death associated with IL-6-induced mitochondrial fragmentation. Shock.

[11] Morley JE, Thomas DR, Wilson MM (2006). Cachexia: Pathophysiology and clinical relevance. Am. J. Clin. Nutr..

[12] Attaix D, Combaret L, Béchet D, Taillandier D (2008). Role of the ubiquitin-proteasome pathway in muscle atrophy in cachexia. Curr. Opin. Support. Palliat. Care.

[13] Bonaldo P, Sandri M (2013). Cellular and molecular mechanisms of muscle atrophy. Dis. Model. Mech..

[14] Hwang PF, Porterfield N, Pannell D, Davis TA, Elster EA (2011). Trauma is danger. J. Transl. Med..

[15] Naglova H, Bucova M (2012). HMGB1 and its physiological and pathological roles. Bratisl. Lek. Listy.

[16] Lotze MT, Tracey KJ (2005). High-mobility group box 1 protein (HMGB1): Nuclear weapon in the immune arsenal. Nat. Rev. Immunol..

[17] Muire PJ, Schwacha MG, Wenke JC (2021). Systemic T cell exhaustion dynamics is linked to early high mobility group box protein 1 (HMGB1) driven hyper-inflammation in a polytrauma rat model. Cells.

[18] Lantos J (2010). Burn trauma induces early HMGB1 release in patients: Its correlation with cytokines. Shock.

[19] Kokkola R (2003). Successful treatment of collagen-induced arthritis in mice and rats by targeting extracellular high mobility group box chromosomal protein 1 activity. Arthritis Rheum..

[20] Muire PJ, Avila JJ, Lofgren AL, Wenke JC (2022). Neutralization of HMGB1 improves fracture healing and gammadelta T lymphocyte counts at the fracture site in a polytrauma rat model. J. Exp. Orthop..

[21] Walker HL, Mason AD (1968). A standard animal burn. J. Trauma.

[22] Gouma E (2012). A simple procedure for estimation of total body surface area and determination of a new value of Meeh's constant in rats. Lab. Anim..

[23] Charles JP, Cappellari O, Spence AJ, Hutchinson JR, Wells DJ (2016). Musculoskeletal geometry, muscle architecture and functional specialisations of the mouse hindlimb. PLoS ONE.

[24] Wan X, Chowdhury IH, Jie Z, Choudhuri S, Garg NJ (2019). Origin of monocytes/macrophages contributing to chronic inflammation in chagas disease: SIRT1 inhibition of FAK-NFkappaB-dependent proliferation and proinflammatory activation of macrophages. Cells.

[25] Lokugamage N, Choudhuri S, Davies C, Chowdhury IH, Garg NJ (2020). Antigen-based nano-immunotherapy controls parasite persistence, inflammatory and oxidative stress, and cardiac fibrosis, the hallmarks of chronic Chagas cardiomyopathy, in a mouse model of *Trypanosoma cruzi* infection. Vaccines (Basel).

[26] Choudhuri S, Garg NJ (2019). PARP1-cGAS-NFkB pathway of proinflammatory macrophage activation by extracellular vesicles released during *Trypanosoma cruzi* infection and Chagas disease. PLoS Pathog..

[27] Haberecht-Müller S, Krüger E, Fielitz J (2021). Out of control: The role of the ubiquitin proteasome system in skeletal muscle during inflammation. Biomolecules.

[28] Asfour HA, Allouh MZ, Said RS (2018). Myogenic regulatory factors: The orchestrators of myogenesis after 30 years of discovery. Exp. Biol. Med. (Maywood).

[29] Chun Y, Kim J (2018). Autophagy: An essential degradation program for cellular homeostasis and life. Cells.

[30] Andersson U, Tracey KJ (2011). HMGB1 is a therapeutic target for sterile inflammation and infection. Annu. Rev. Immunol..

[31] Jeschke MG (2011). Long-term persistance of the pathophysiologic response to severe burn injury. PLoS ONE.

[32] Matsushima A (2004). Early activation of gammadelta T lymphocytes in patients with severe systemic inflammatory response syndrome. Shock.

[33] Soskic B (2020). CD80 on human T cells is associated with FoxP3 expression and supports Treg homeostasis. Front. Immunol..

[34] Knuth CM, Auger C, Jeschke MG (2021). Burn-induced hypermetabolism and skeletal muscle dysfunction. Am. J. Physiol. Cell Physiol..

[35] Taverna S (2022). High mobility group box 1: Biological functions and relevance in oxidative stress related chronic diseases. Cells.

[36] Peltz ED (2009). HMGB1 is markedly elevated within 6 h of mechanical trauma in humans. Shock.

[37] Saeman MR, DeSpain K, Liu MM, Wolf SE, Song J (2016). Severe burn increased skeletal muscle loss in mdx mutant mice. J. Surg. Res..

[38] Burckart K, Beca S, Urban RJ, Sheffield-Moore M (2010). Pathogenesis of muscle wasting in cancer cachexia: Targeted anabolic and anticatabolic therapies. Curr. Opin. Clin. Nutr. Metab. Care.

[39] Song J, de Libero J, Wolf SE (2014). Hepatic autophagy after severe burn in response to endoplasmic reticulum stress. J. Surg. Res..

[40] Yang X, Xue P, Liu X, Xu X, Chen Z (2018). HMGB1/autophagy pathway mediates the atrophic effect of TGF-beta1 in denervated skeletal muscle. Cell Commun. Signal.

[41] Sandri M (2013). Protein breakdown in muscle wasting: Role of autophagy-lysosome and ubiquitin-proteasome. Int. J. Biochem. Cell Biol..

[42] Muth IE (2015). HMGB1 and RAGE in skeletal muscle inflammation: Implications for protein accumulation in inclusion body myositis. Exp. Neurol..

[43] De Mori R (2007). Multiple effects of high mobility group box protein 1 in skeletal muscle regeneration. Arterioscler. Thromb. Vasc. Biol..

[44] Riuzzi F, Sorci G, Sagheddu R, Donato R (2012). HMGB1-RAGE regulates muscle satellite cell homeostasis through p38-MAPK- and myogenin-dependent repression of Pax7 transcription. J. Cell Sci..

[45] Li L (2021). Pharmacological inhibition of HMGB1 prevents muscle wasting. Front. Pharmacol..

[46] Wen W (2022). Alcohol Induces Zebrafish Skeletal Muscle Atrophy through HMGB1/TLR4/NF-kappaB Signaling. Life (Basel).

[47] Heideman M, Bengtsson A (1992). The immunologic response to thermal injury. World J. Surg..

[48] Valvis SM, Waithman J, Wood FM, Fear MW, Fear VS (2015). The immune response to skin trauma is dependent on the etiology of injury in a mouse model of burn and excision. J. Invest. Dermatol..

[49] Moins-Teisserenc H (2021). Severe altered immune status after burn injury is associated with bacterial infection and septic shock. Front. Immunol..

[50] Corrick KL (2015). Serum from human burn victims impairs myogenesis and protein synthesis in primary myoblasts. Front Physiol.

[51] Lee SA, Kwak MS, Kim S, Shin JS (2014). The role of high mobility group box 1 in innate immunity. Yonsei Med. J..

[52] Careccia G (2021). Rebalancing expression of HMGB1 redox isoforms to counteract muscular dystrophy. Sci. Transl. Med..

