# Supplementary material for: Acute muscle mass loss was alleviated with HMGB1 neutralizing antibody treatment in severe burned rats
Source: Sci Rep. 2023 Jun 24;13:10250. doi: 10.1038/s41598-023-37476-4 (PMC10290662; doi:10.1038/s41598-023-37476-4)
Supplement: Supplementary file 1 — Supplementary Legends. [file 41598_2023_37476_MOESM1_ESM.docx]

**Supplemental figure legend**

**S1 Figure. Fluorescence-minus-one (FMO) controls**. Single cell suspensions of RAT peripheral blood mononuclear cells (PBMCs) and splenocytes were labeled with fluorescent-conjugated antibodies for surface molecules and intracellular cytokines and acquired on a BD LSR Fortessa. Markers for granulocyte (granulocyte marker), αβ, γδ, CD4, CD62L, CD127, TNFα, IFNγ, CD80, and CD86 were used in the T cell panel. To define the lower limit of the gate for each molecule, all markers in the panel were done excluding only the fluor of the marker of interest (e.g., a granulocyte FMO is stained for all markers except the granulocyte antibody). Positive and negative sub-populations of the cells were captured using the FMOs of the markers.

**S2 Figure. Western blot raw images** including 2.1) HMGB1; 2.2) Ubiquitin; 2.3) MURF-1; 2.4) Caspase-3; 2.5) PCNA; 2.6) Myogenin; 2.7) GAPDH; 2.8) Atg-3; 2.9) Atg-5; 2.10) Atg-7; 2.11) Atg-12; 2.12) Beclin-1; 2.13) LC3A/B; 2.14) Cyt-C; 2.15) Hsp60; 2.16) GAPDH.

**Supplemental Tables**

**Table S1: Flow cytometry antibodies.**

**Table S2: Antibodies used for Western blotting**

**Table S3. Detailed flow cytometry analysis data.** Sprague Dawley rats were subjected to sham burn or 30% TBSA burn, treated with one dose of vehicle or anti-HMGB1 antibody and euthanized 3 days later. Single cell suspensions of bone marrow cells, peripheral blood mononuclear cells (PBMCs), and splenocytes were labeled with fluorescent-conjugated antibodies and analyzed by flow cytometry. Data were tested by D’Agostino & Pearson Omnibus Normality test. Datasets were analyzed by 1-way analysis of variance (ANOVA) followed by Tukey’s post-hoc test or Kruskal-Wallis H/Dunn’s post-hoc test. Data are presented as mean values ± SEM and derived from n ≥ 6 rats per group. Significance is annotated with * Sham vs Burn + Vehicle or Burn + HMGB1 Ab and ^ Burn + Vehicle vs Burn + HMGB1 Ab, and p values of <0.05, <0.01, and <0.001 are presented with one, two, and three symbols, respectively**.**
