# Supplementary figures and images for: Acute muscle mass loss was alleviated with HMGB1 neutralizing antibody treatment in severe burned rats
Source: Sci Rep. 2023 Jun 24;13:10250. doi: 10.1038/s41598-023-37476-4 (PMC10290662; doi:10.1038/s41598-023-37476-4)

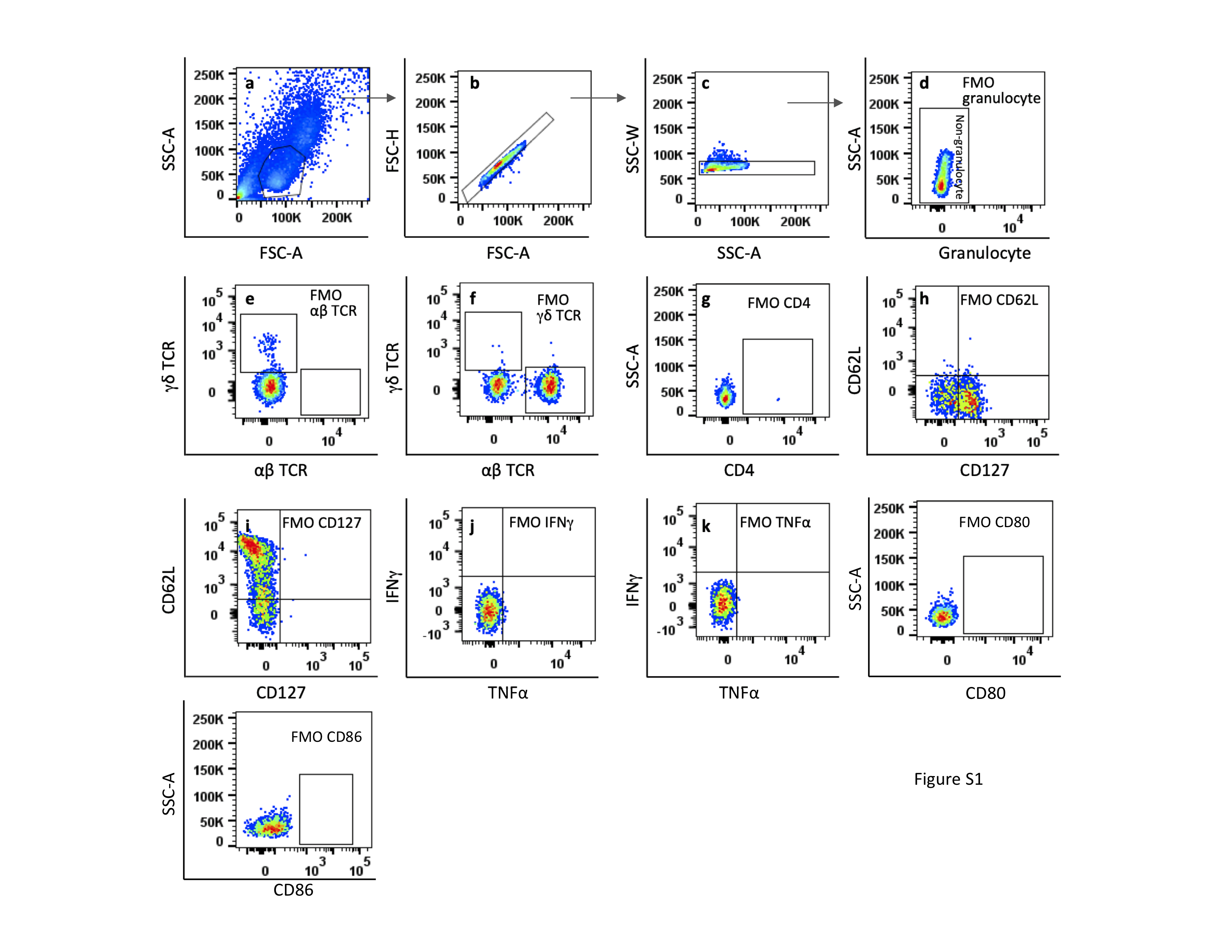

Supplement: Supplementary file 2 — Supplementary Figure S1. [file 41598_2023_37476_MOESM2_ESM.tif]
