# Supplementary material for: Acute muscle mass loss was alleviated with HMGB1 neutralizing antibody treatment in severe burned rats
Source: Sci Rep. 2023 Jun 24;13:10250. doi: 10.1038/s41598-023-37476-4 (PMC10290662; doi:10.1038/s41598-023-37476-4)
Supplement: Supplementary file 3 — Supplementary Figure S2. [file 41598_2023_37476_MOESM3_ESM.pdf]

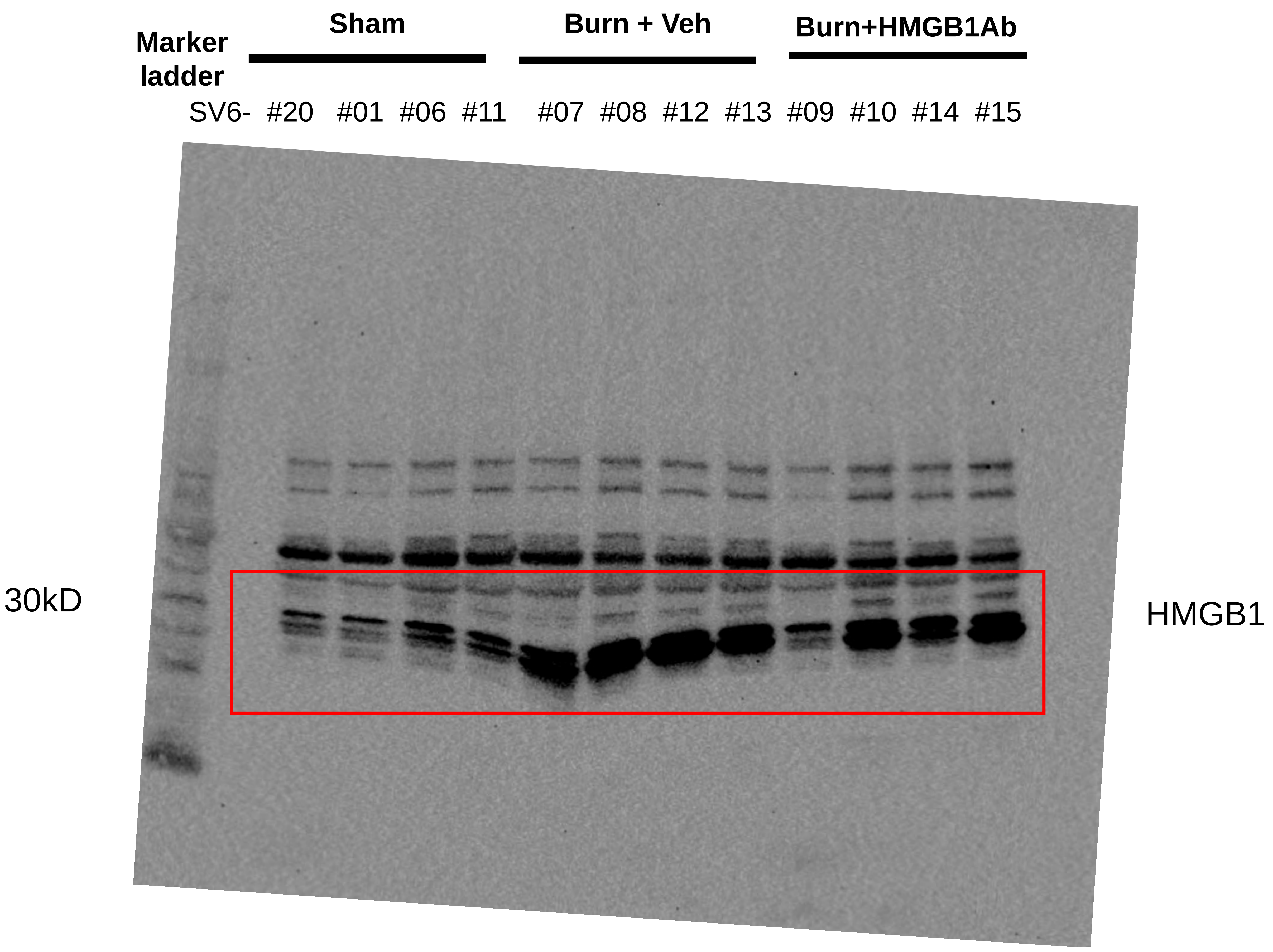

Supplemental figure 2.1 for figure 4a: Western blot raw image of HMGB1 expression in muscle tissue from sham burn rats (Sham), or burn rats with vehicle treatment (Burn+Veh) and with HMGB1 antibody treatment (Burn+HMGB1) at day 3 (n=4/each group). The 1st lane is protein marker ladder, following with 12 20µg of protein lysate samples extracted from labeled individual animal in the study (SV6-). A red box circled HMGB1 protein band at the range of 30kD.

[file name:L-a#01-HMGB1-SongJ 2022-04-25 10h04m27s-c-hmgb1(Chemiluminescence).tif]

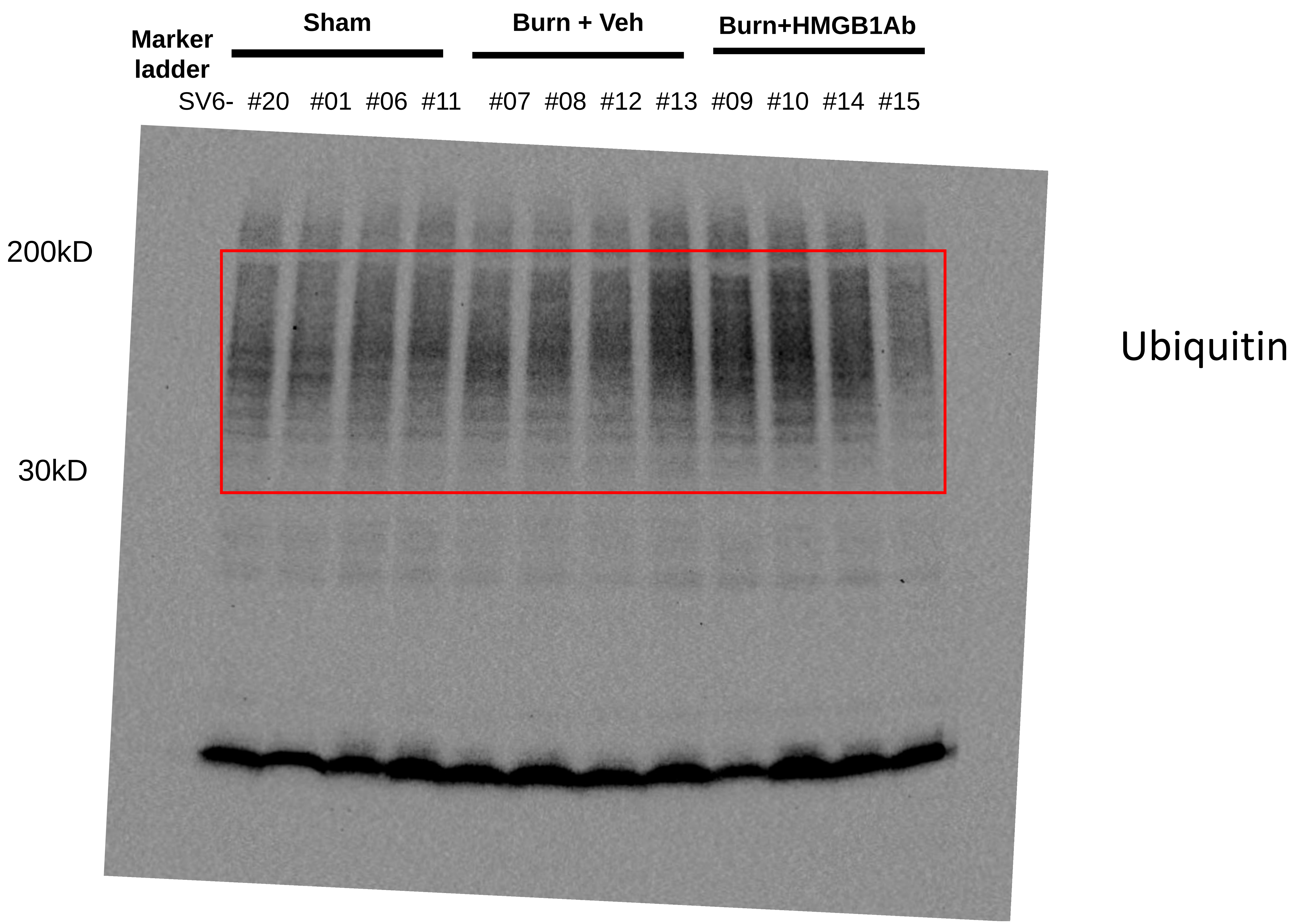

Supplemental figure 2.2 for figure 4a: Western blot raw image of Ubiquitin expression in muscle tissue from sham burn rats (Sham), or burn rats with vehicle treatment (Burn+Veh) and with HMGB1 antibody treatment (Burn+HMGB1) at day 3 (n=4/each group). The 1st lane is protein marker ladder, following with 12 20µg of protein lysate samples extracted from labeled individual animal in the study (SV6-). A red box circled Ubiquitin smear protein bands between the range of 200 to 30 kD .

[file name:L-a#02-Ubiqitin-SongJ 2022-10-06 08h57m48s-a-ubqutin(Chemiluminescence).tif]

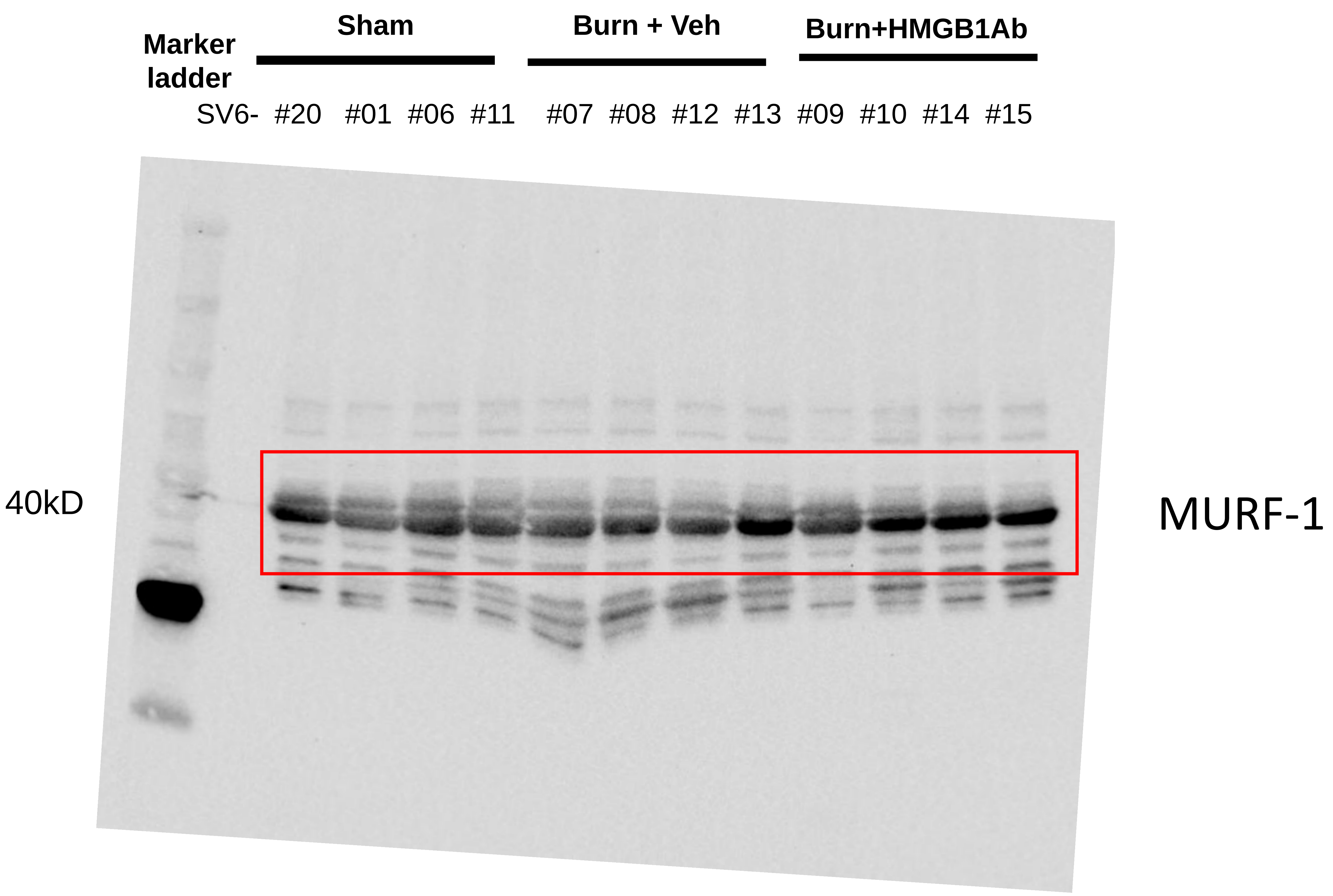

Supplemental figure 2.3 for figure 4a: Western blot raw image of MURF-1 expression in muscle tissue from sham burn rats (Sham), or burn rats with vehicle treatment (Burn+Veh) and with HMGB1 antibody treatment (Burn+HMGB1) at day 3 (n=4/each group). The 1st lane is protein marker ladder, following with 12 20µg of protein lysate samples extracted from labeled individual animal in the study (SV6-). A red box circled MURF-1 protein bands at the range of 40kD .

[file name:L-a#03-Murff1-SongJ 2022-04-26 09h59m14s-c-murf(Chemiluminescence).tif]

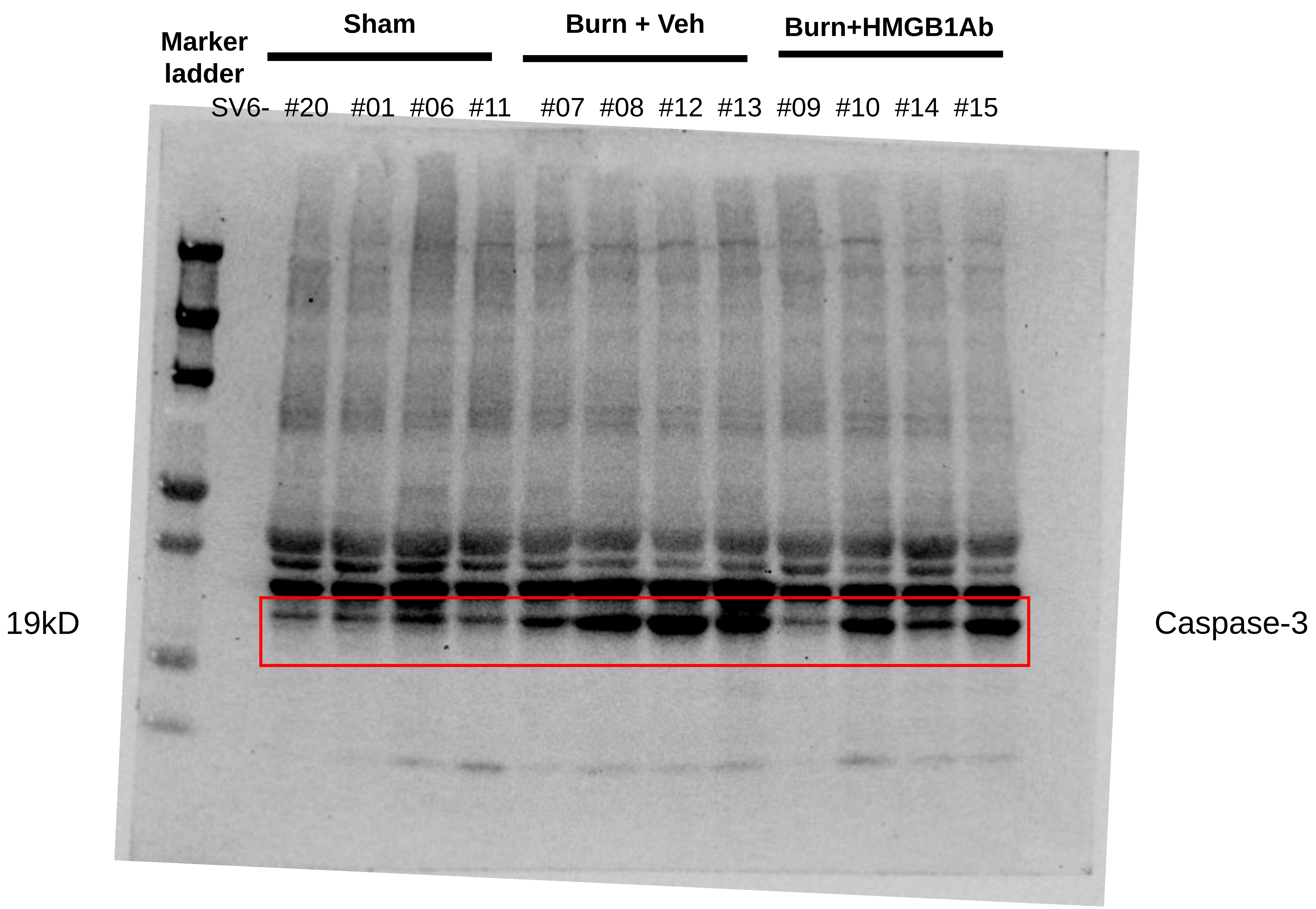

Supplemental figure 2.4 for figure 4a: Western blot raw image of cleaved caspase-3 expression in muscle tissue from sham burn rats (Sham), or burn rats with vehicle treatment (Burn+Veh) and with HMGB1 antibody treatment (Burn+HMGB1) at day 3 (n=4/each group). The 1st lane is protein marker ladder, following with 12 20µg of protein lysate samples extracted from labeled individual animal in the study (SV6-). A red box circled caspase-3 protein bands at the range of 19kD .

[file name:L-a#04-caspsase3-SongJ 2022-04-25 10h41m14s-sv6-a-caspase3(Chemiluminescence).tif]

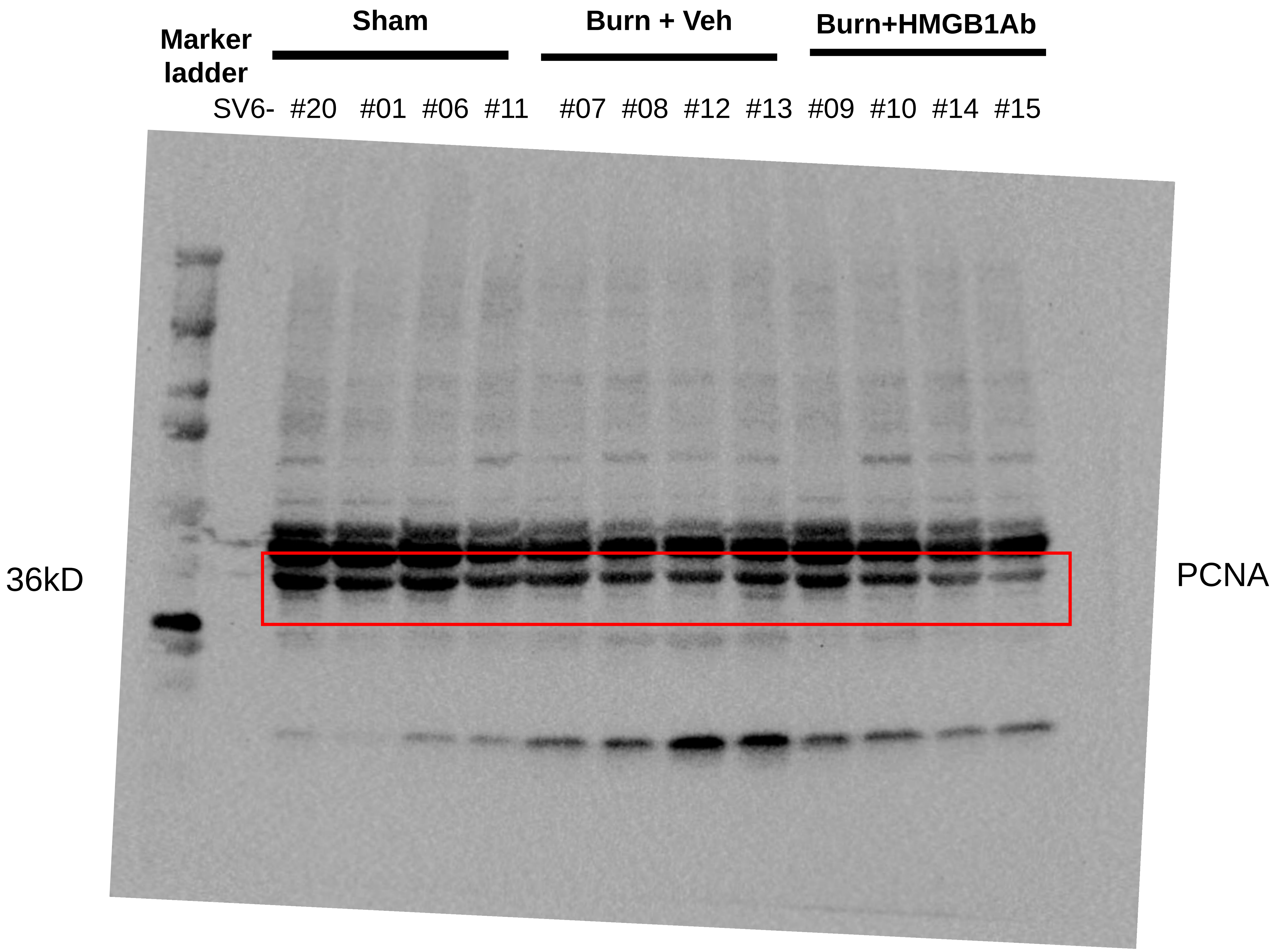

Supplemental figure 2.5 for figure 4a: Western blot raw image of PCNA expression in muscle tissue from sham burn rats (Sham), or burn rats with vehicle treatment (Burn+Veh) and with HMGB1 antibody treatment (Burn+HMGB1) at day 3 (n=4/each group). The 1st lane is protein marker ladder, following with 12 20µg of protein lysate samples extracted from labeled individual animal in the study (SV6-). A red box circled PCNA protein bands at the range of 36kD .

[file name:L-a#05-PCNA-SongJ 2022-04-28 09h43m21s-d-pcna(Chemiluminescence).tif]

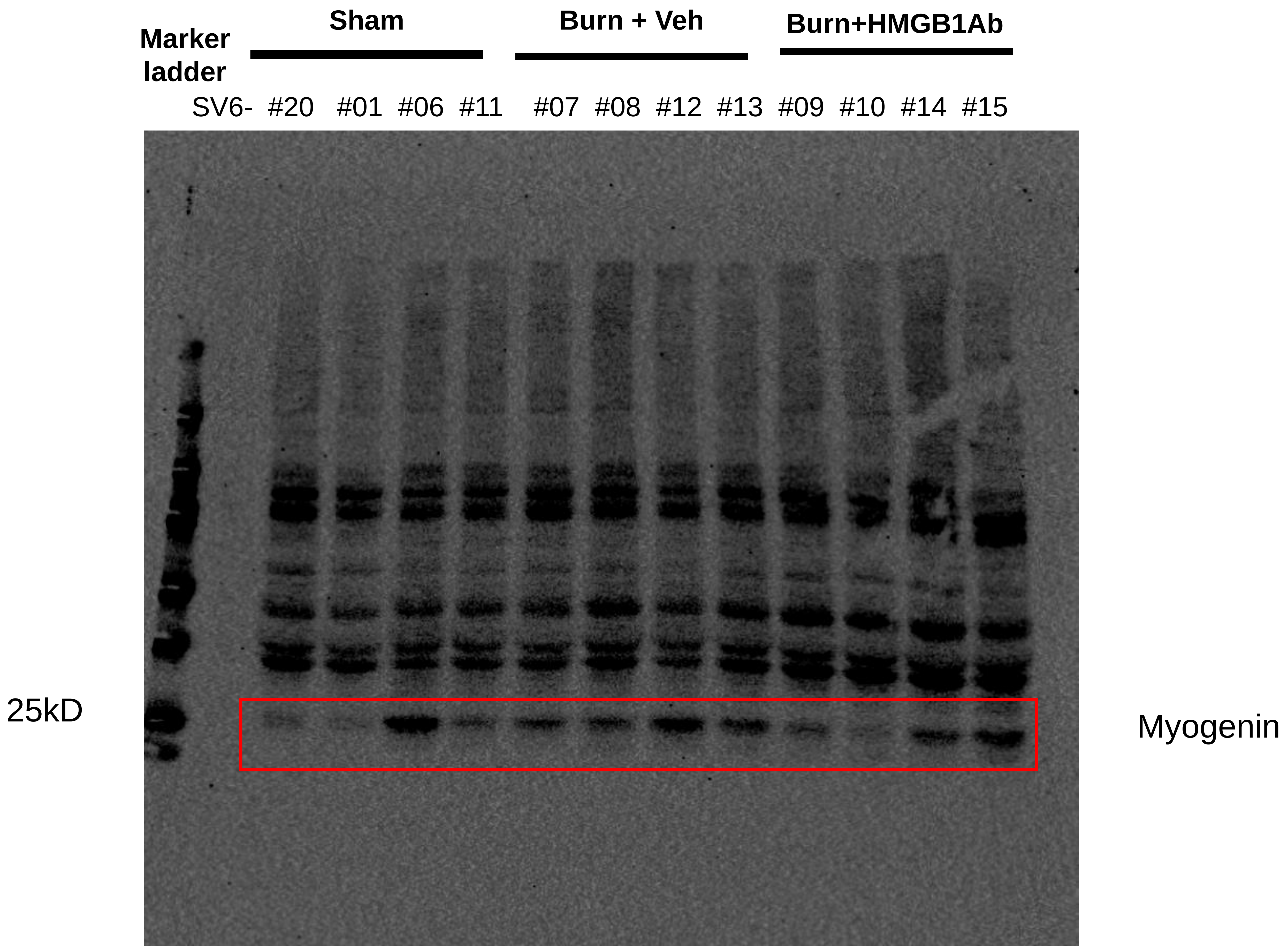

Supplemental figure 2.6 for figure 4a: Western blot raw image of Myogenin expression in muscle tissue from sham burn rats (Sham), or burn rats with vehicle treatment (Burn+Veh) and with HMGB1 antibody treatment (Burn+HMGB1) at day 3 (n=4/each group). The 1st lane is protein marker ladder, following with 12 20µg of protein lysate samples extracted from labeled individual animal in the study (SV6-). A red box circled myogenin protein bands at the range of 25kD .

[file name: L-a#06-Myogenin-SongJ 2022-10-03 10h26m15s-c-myg(Chemiluminescence).tif]

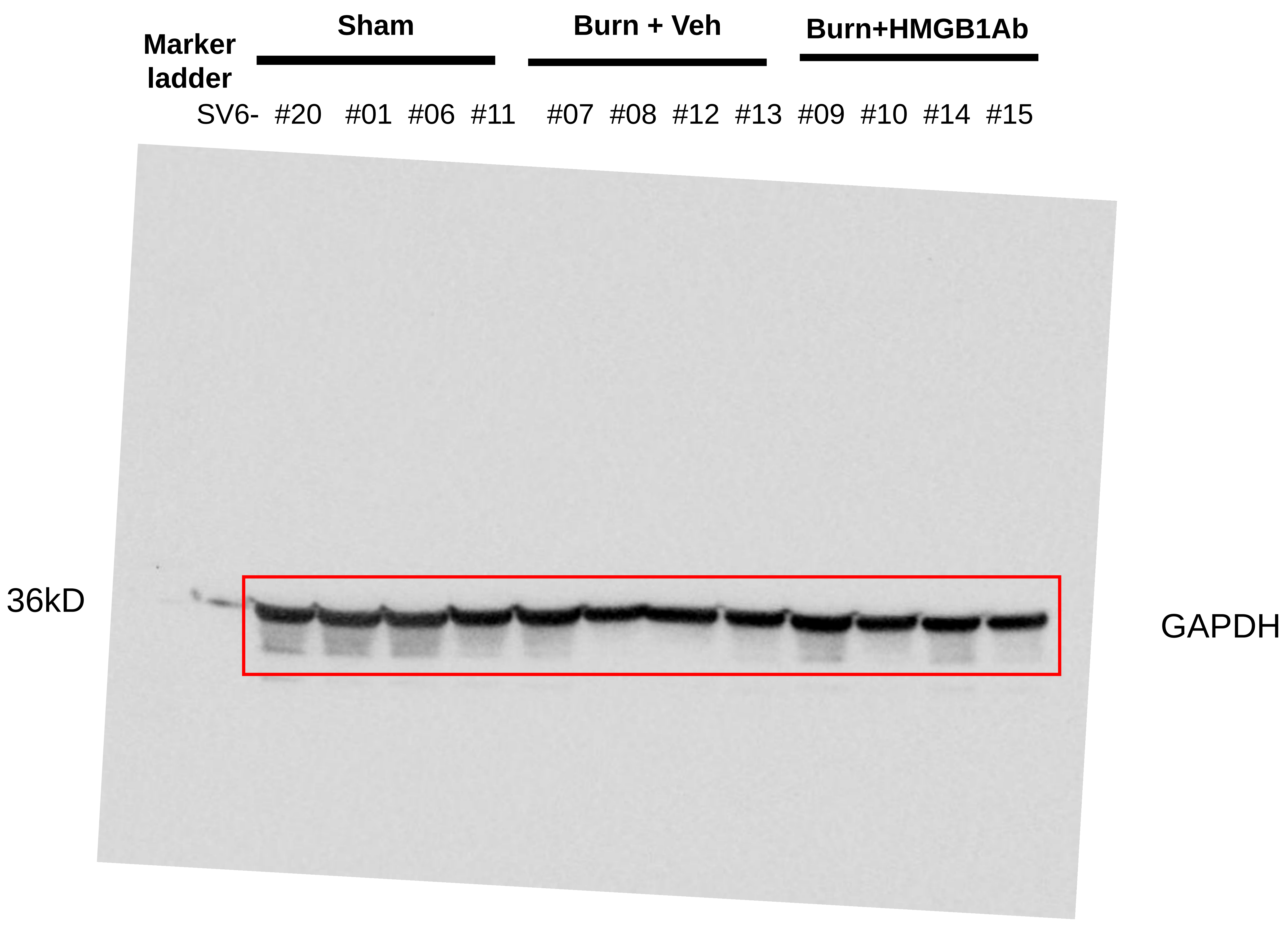

Supplemental figure 2.7 for figure 4a: Western blot raw image of GAPDH expression in muscle tissue from sham burn rats (Sham), or burn rats with vehicle treatment (Burn+Veh) and with HMGB1 antibody treatment (Burn+HMGB1) at day 3 (n=4/each group). The 1st lane is protein marker ladder, following with 12 20µg of protein lysate samples extracted from labeled individual animal in the study (SV6-). A red box circled GAPDH protein bands at the range of 36kD .

[file name: L-a#07GAPDH-SongJ 2022-04-26 09h16m05s-a-desmin-b-gapdh(Chemiluminescence).tif]

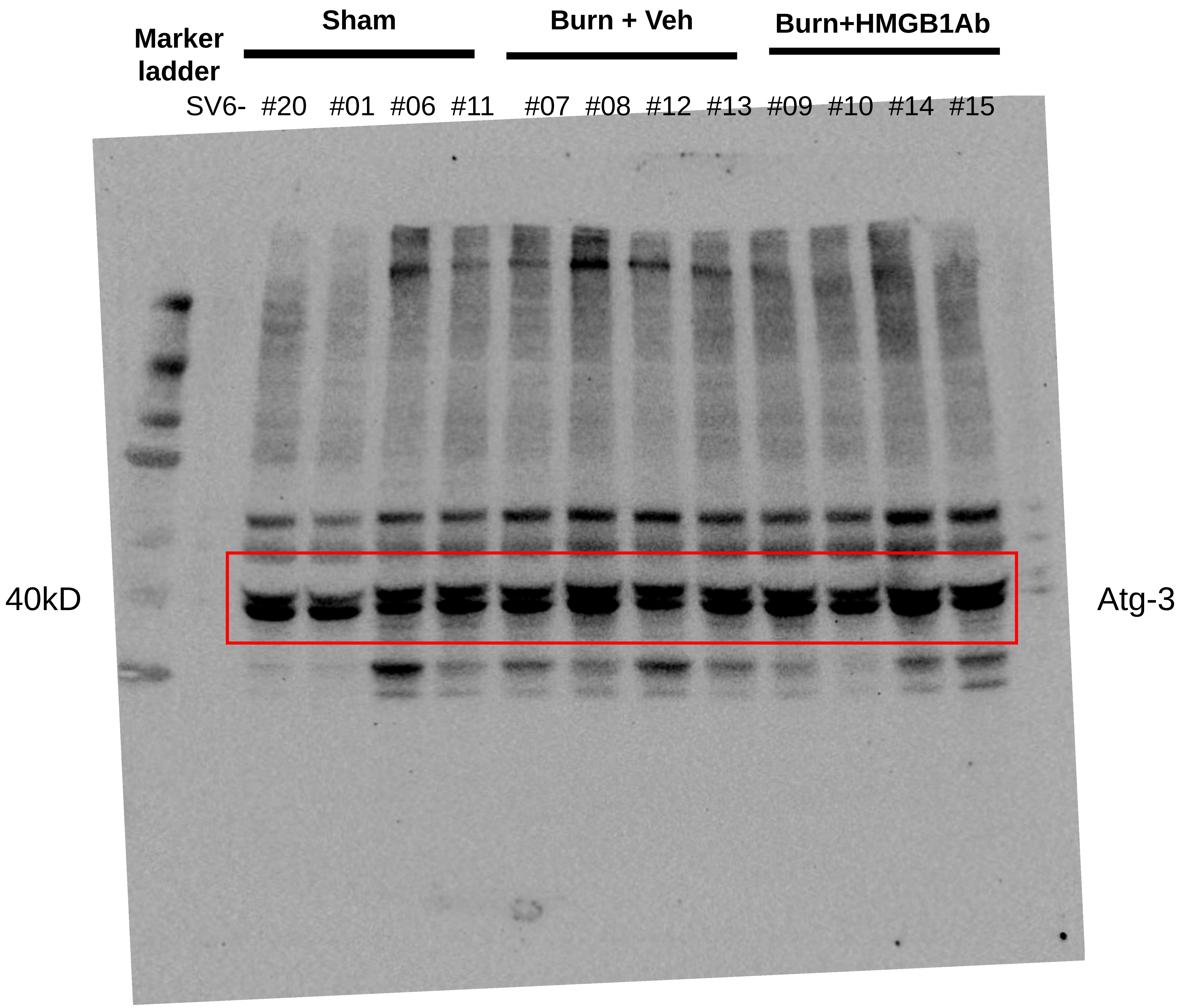

Supplemental figure 2.8 for figure 4h: Western blot raw image of Atg-3 expression in muscle tissue from sham burn rats (Sham), or burn rats with vehicle treatment (Burn+Veh) and with HMGB1 antibody treatment (Burn+HMGB1) at day 3 (n=4/each group). The 1st lane is protein marker ladder, following with 12 20µg of protein lysate samples extracted from labeled individual animal in the study (SV6-). A red box circled Atg-3 protein bands at the range of 40kD .

[file name: L-h#01-Atg3-SongJ 2022-10-04 09h28m35s-d-atg3(Chemiluminescence).tif]

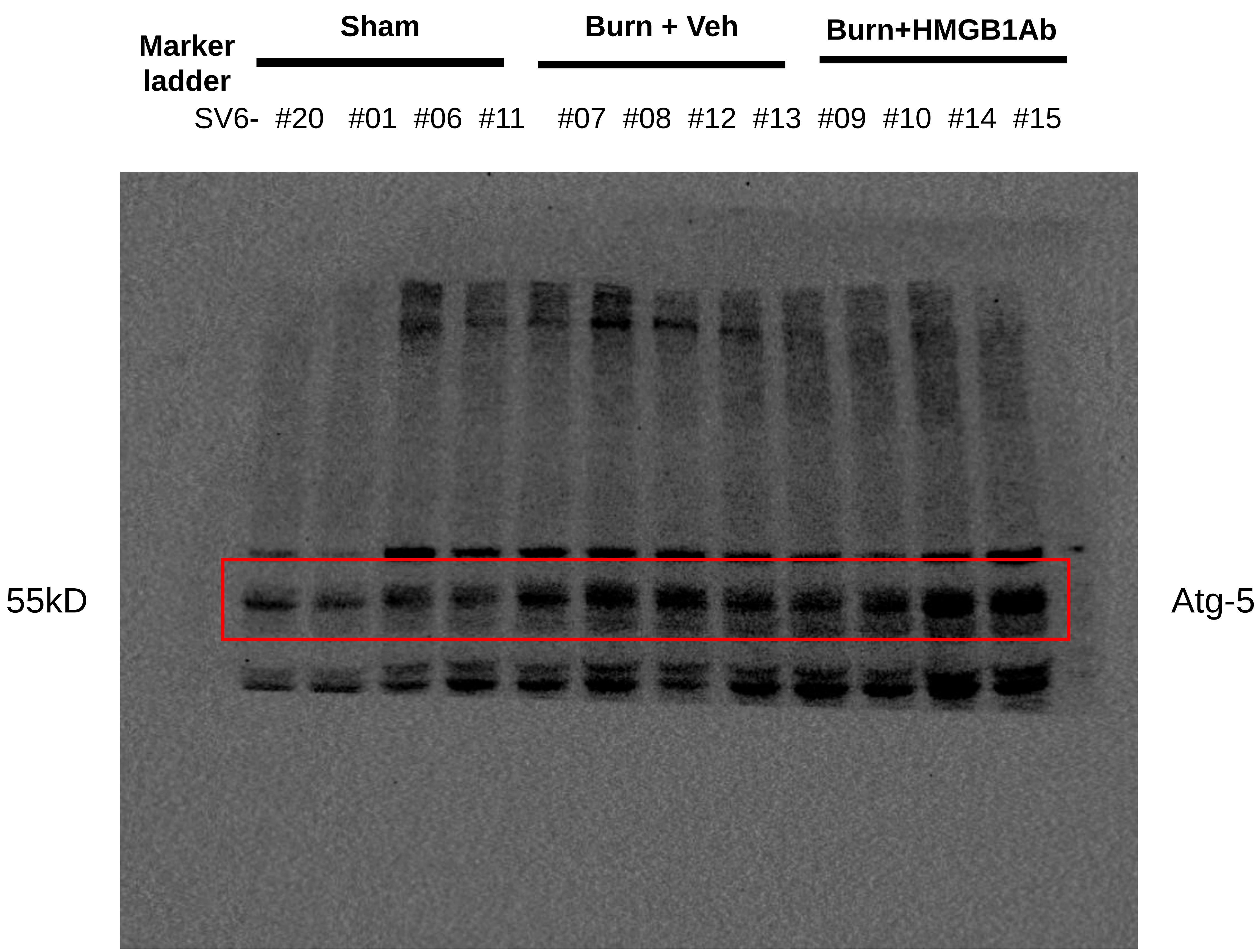

Supplemental figure 2.9 for figure 4h: Western blot raw image of Atg-5 expression in muscle tissue from sham burn rats (Sham), or burn rats with vehicle treatment (Burn+Veh) and with HMGB1 antibody treatment (Burn+HMGB1) at day 3 (n=4/each group). The 1st lane is protein marker ladder, following with 12 20µg of protein lysate samples extracted from labeled individual animal in the study (SV6-). A red box circled Atg-5 protein bands at the range of 55kD .

[file name: L-h#02-Atg5-SongJ 2022-10-05 12h16m32s-d-atg5(Chemiluminescence).tif]

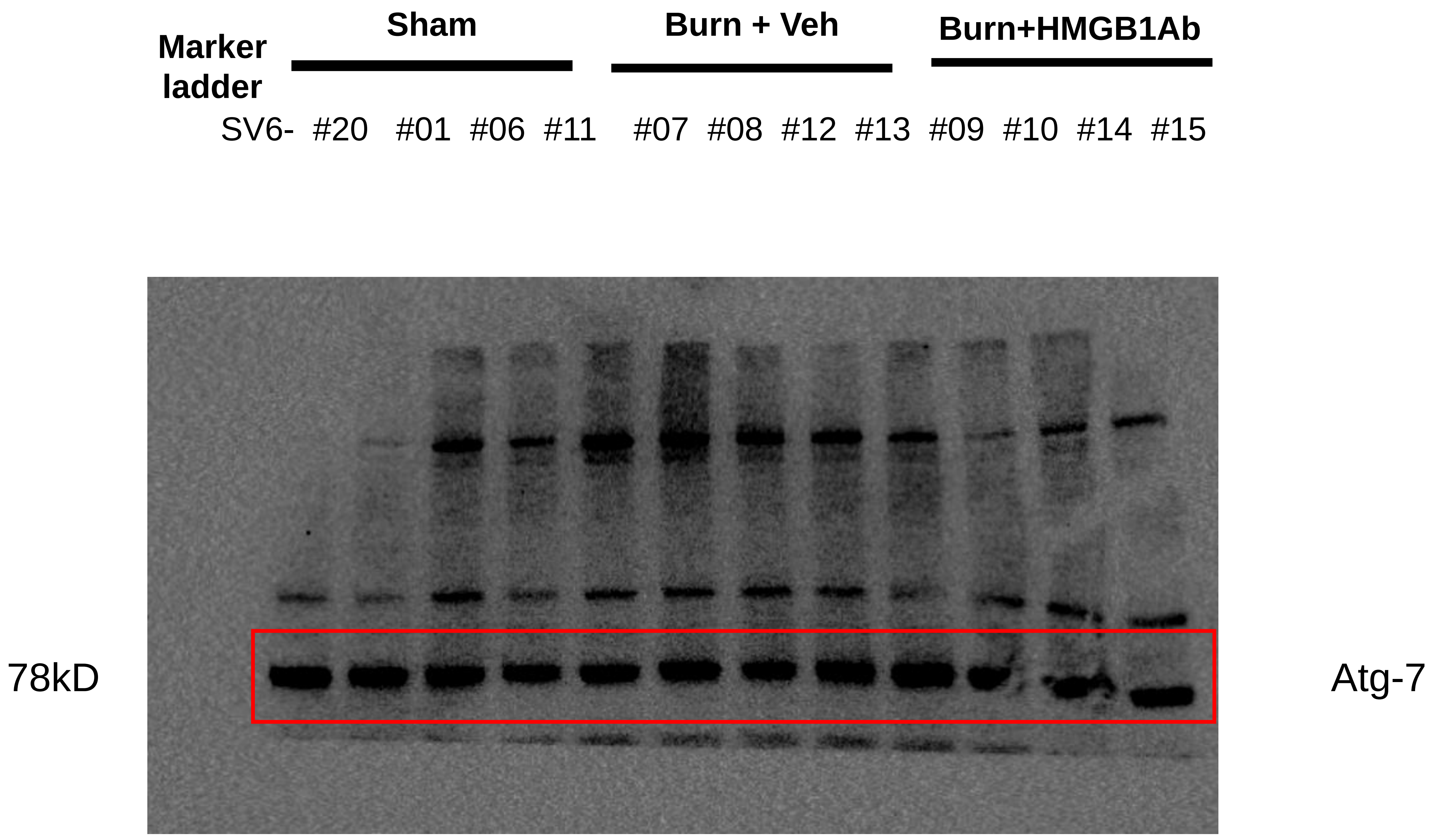

Supplemental figure 2.10 for figure 4h: Western blot raw image of Atg-7 expression in muscle tissue from sham burn rats (Sham), or burn rats with vehicle treatment (Burn+Veh) and with HMGB1 antibody treatment (Burn+HMGB1) at day 3 (n=4/each group). The 1st lane is protein marker ladder, following with 12 20µg of protein lysate samples extracted from labeled individual animal in the study (SV6-). A red box circled Atg-7 protein bands at the range of 78kD .

[file name: L-h#03-Atg7-SongJ 2022-10-05 12h12m33s-c-atg7(Chemiluminescence).tif]

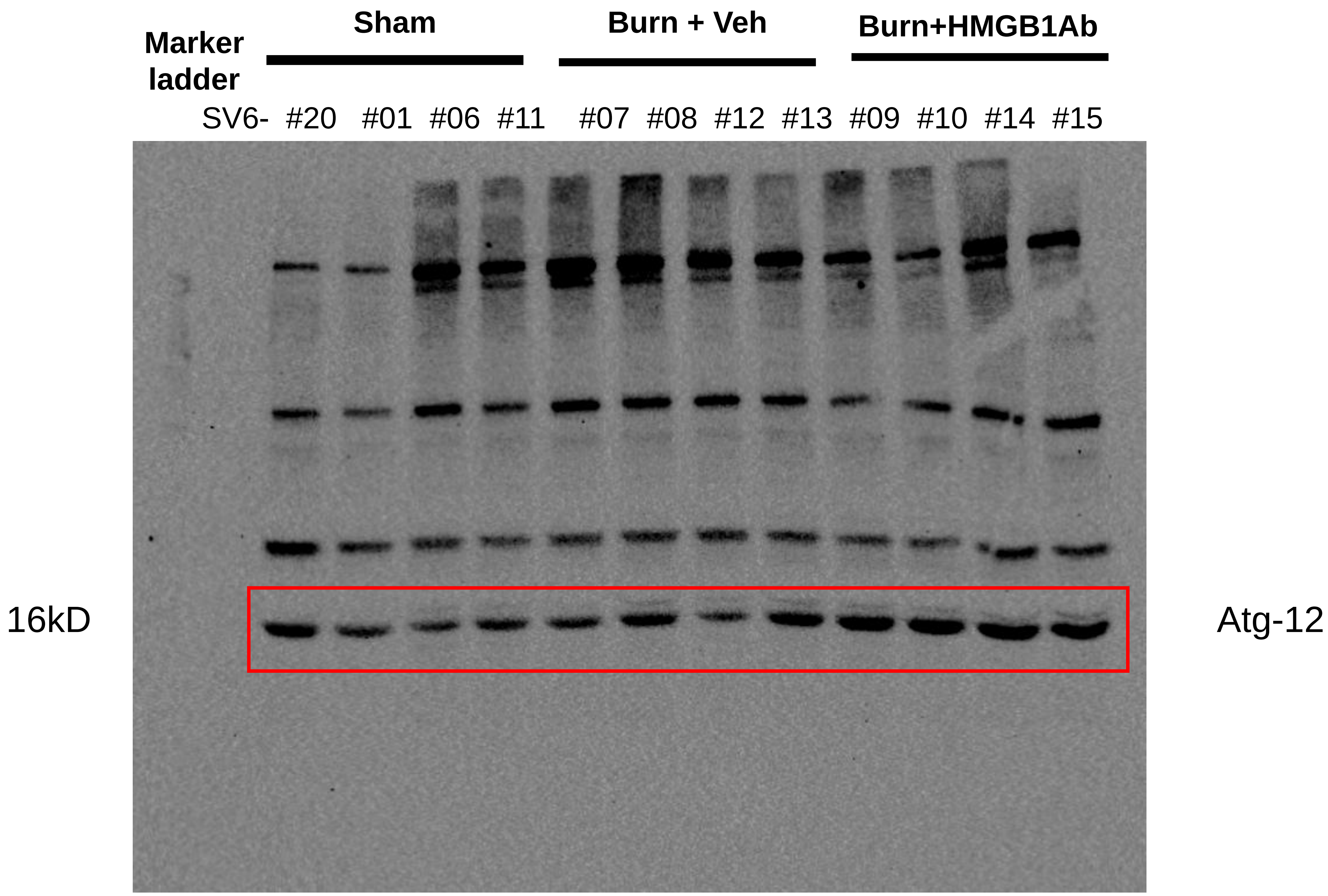

Supplemental figure 2.11 for figure 4h: Western blot raw image of Atg-12 expression in muscle tissue from sham burn rats (Sham), or burn rats with vehicle treatment (Burn+Veh) and with HMGB1 antibody treatment (Burn+HMGB1) at day 3 (n=4/each group). The 1st lane is protein marker ladder, following with 12 20µg of protein lysate samples extracted from labeled individual animal in the study (SV6-). A red box circled Atg-12 protein bands at the range of 16kD .

[file name: L-h#04-Atg12-SongJ 2022-10-04 09h23m38s-c-atg12(Chemiluminescence).tif]

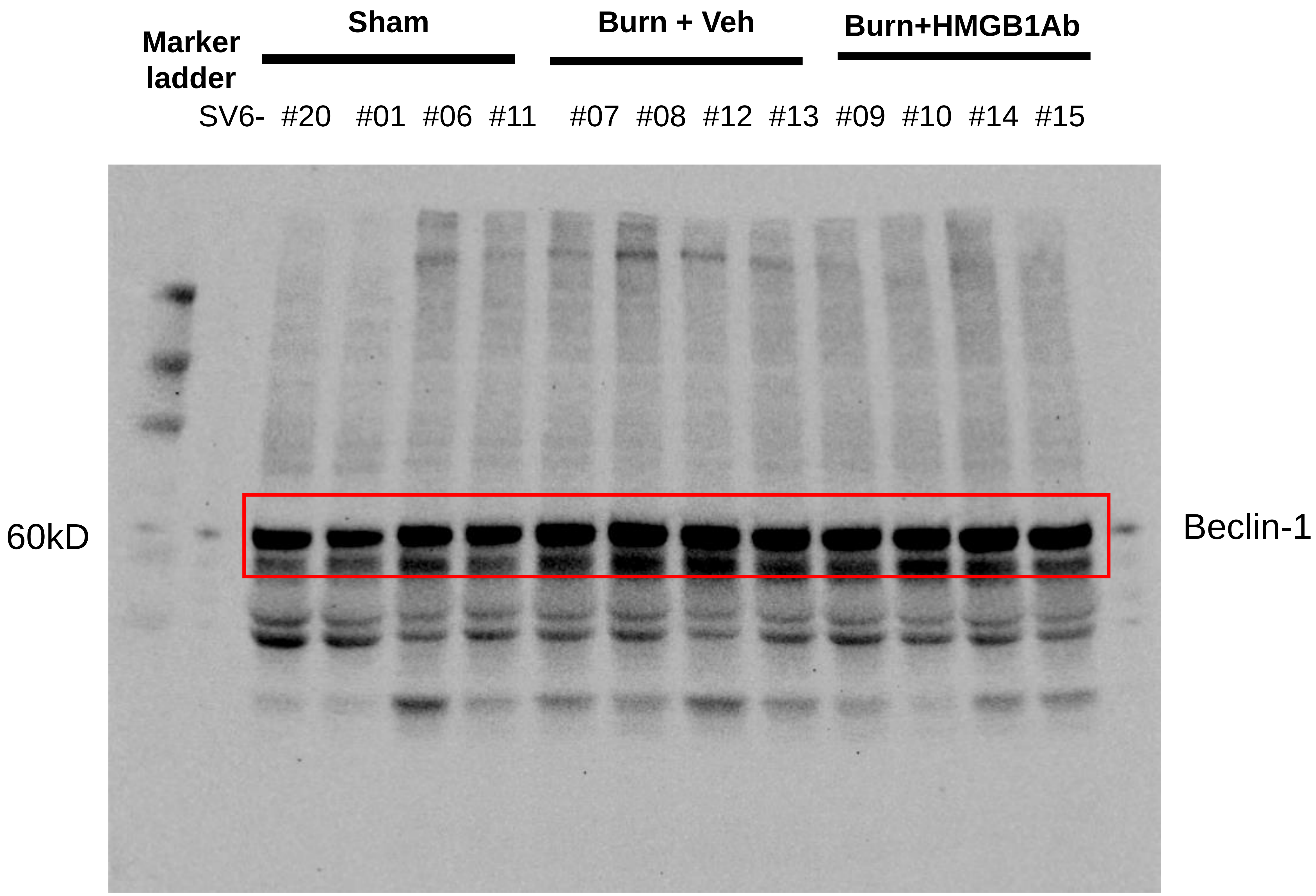

Supplemental figure 2.12 for figure 4h: Western blot raw image of Beclin-1 expression in muscle tissue from sham burn rats (Sham), or burn rats with vehicle treatment (Burn+Veh) and with HMGB1 antibody treatment (Burn+HMGB1) at day 3 (n=4/each group). The 1st lane is protein marker ladder, following with 12 20µg of protein lysate samples extracted from labeled individual animal in the study (SV6-). A red box circled Beclin-1 protein bands at the range of 60kD .

[file name: L-h#05-Beclin1-SongJ 2022-10-03 10h17m54s-beclin1-d(Chemiluminescence).tif]

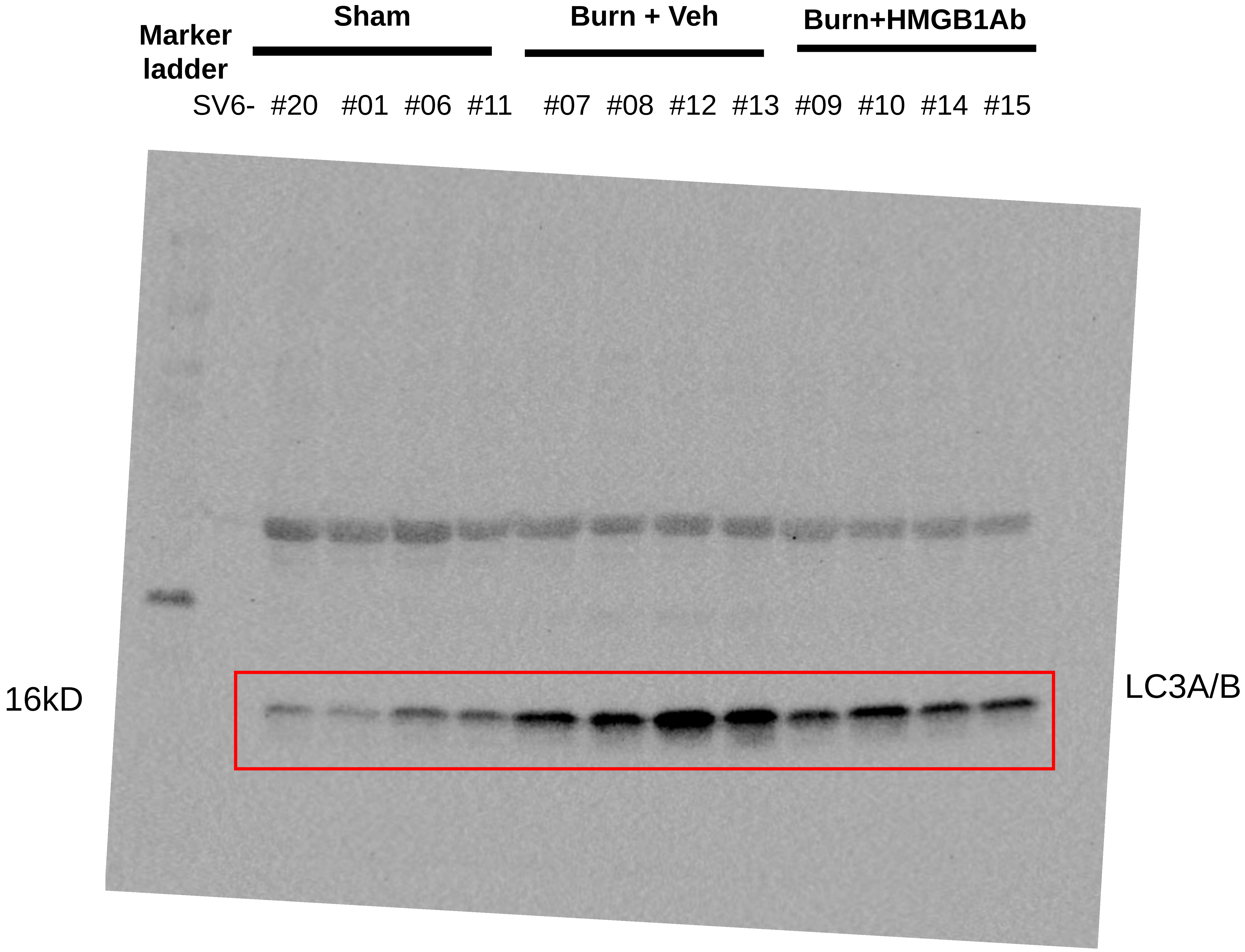

Supplemental figure 2.13 for figure 4h: Western blot raw image of LC3A/B expression in muscle tissue from sham burn rats (Sham), or burn rats with vehicle treatment (Burn+Veh) and with HMGB1 antibody treatment (Burn+HMGB1) at day 3 (n=4/each group). The 1st lane is protein marker ladder, following with 12 20µg of protein lysate samples extracted from labeled individual animal in the study (SV6-). A red box circled LC3A/B protein bands at the range of 16kD .

[file name: L-h#06-LC3AB-SongJ 2022-04-26 09h51m53s-d-lc3a-b(Chemiluminescence).tif]

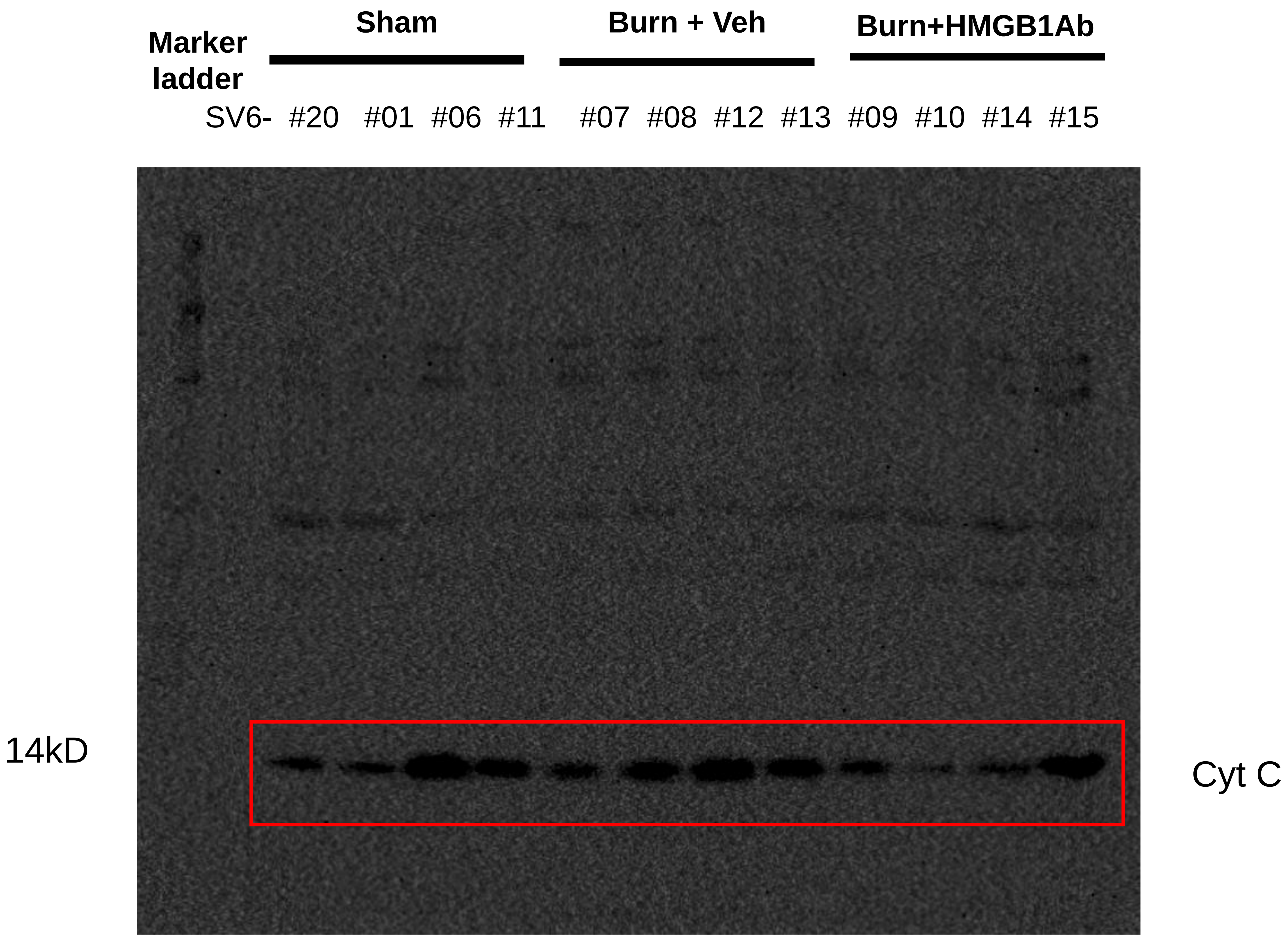

Supplemental figure 2.14 for figure 4h: Western blot raw image of Cytochrome C (CytC) expression in muscle tissue from sham burn rats (Sham), or burn rats with vehicle treatment (Burn+Veh) and with HMGB1 antibody treatment (Burn+HMGB1) at day 3 (n=4/each group). The 1st lane is protein marker ladder, following with 12 20µg of protein lysate samples extracted from labeled individual animal in the study (SV6-). A red box circled Cytochrome C (CytC) protein bands at the range of 14kD .

[file name: L-h#07-CytC-SongJ 2022-10-06 09h03m00s-c-cytu-ochromec(Chemiluminescence).tif]

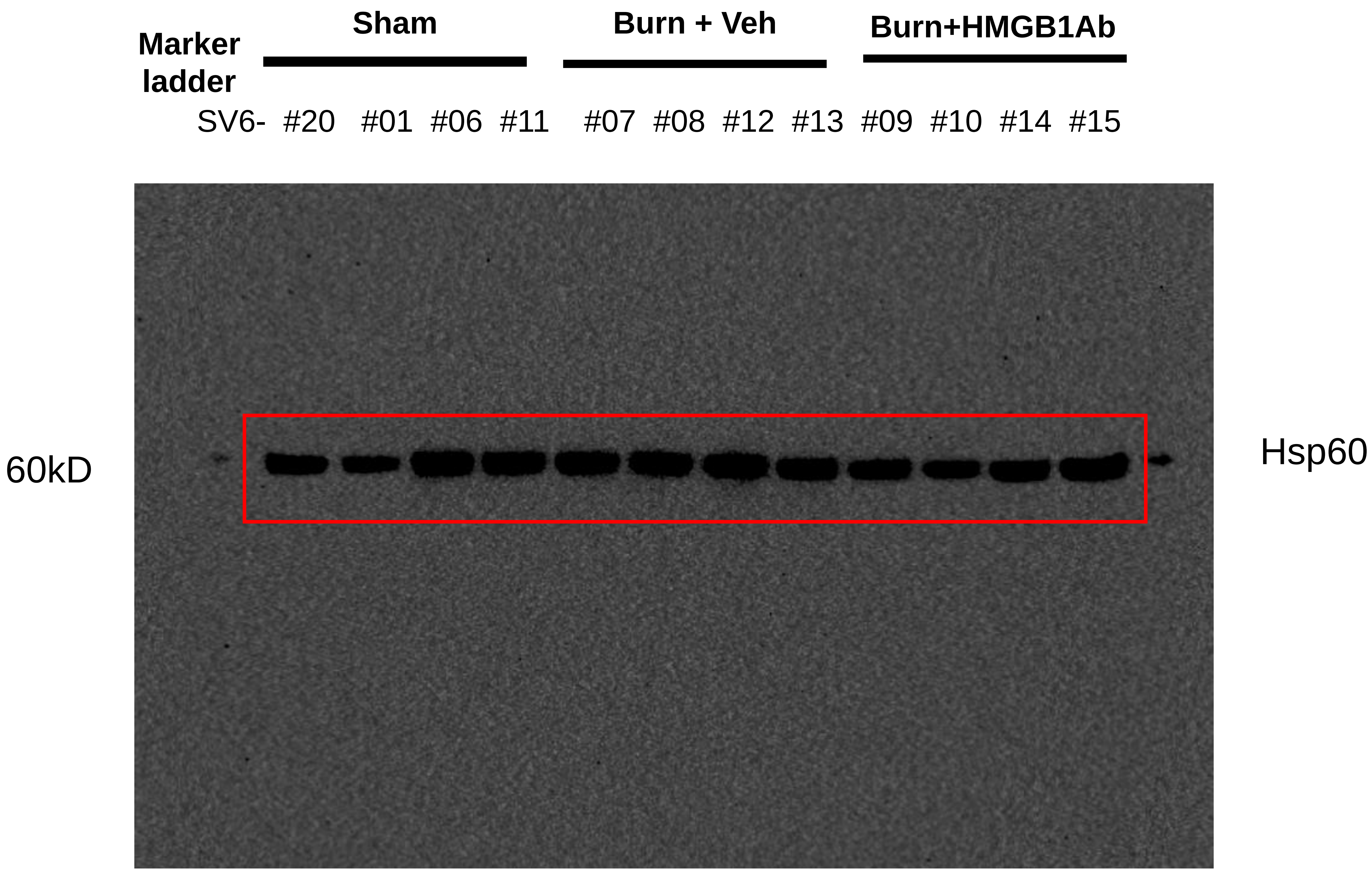

Supplemental figure 2.15 for figure 4h: Western blot raw image of Hsp60expression in muscle tissue from sham burn rats (Sham), or burn rats with vehicle treatment (Burn+Veh) and with HMGB1 antibody treatment (Burn+HMGB1) at day 3 (n=4/each group). The 1st lane is protein marker ladder, following with 12 20µg of protein lysate samples extracted from labeled individual animal in the study (SV6-). A red box circled Hsp60 protein bands at the range of 60kD .

[file name: L-h#08-Hsp60-SongJ 2022-10-06 08h40m30s-d-hsp60(Chemiluminescence).tif]

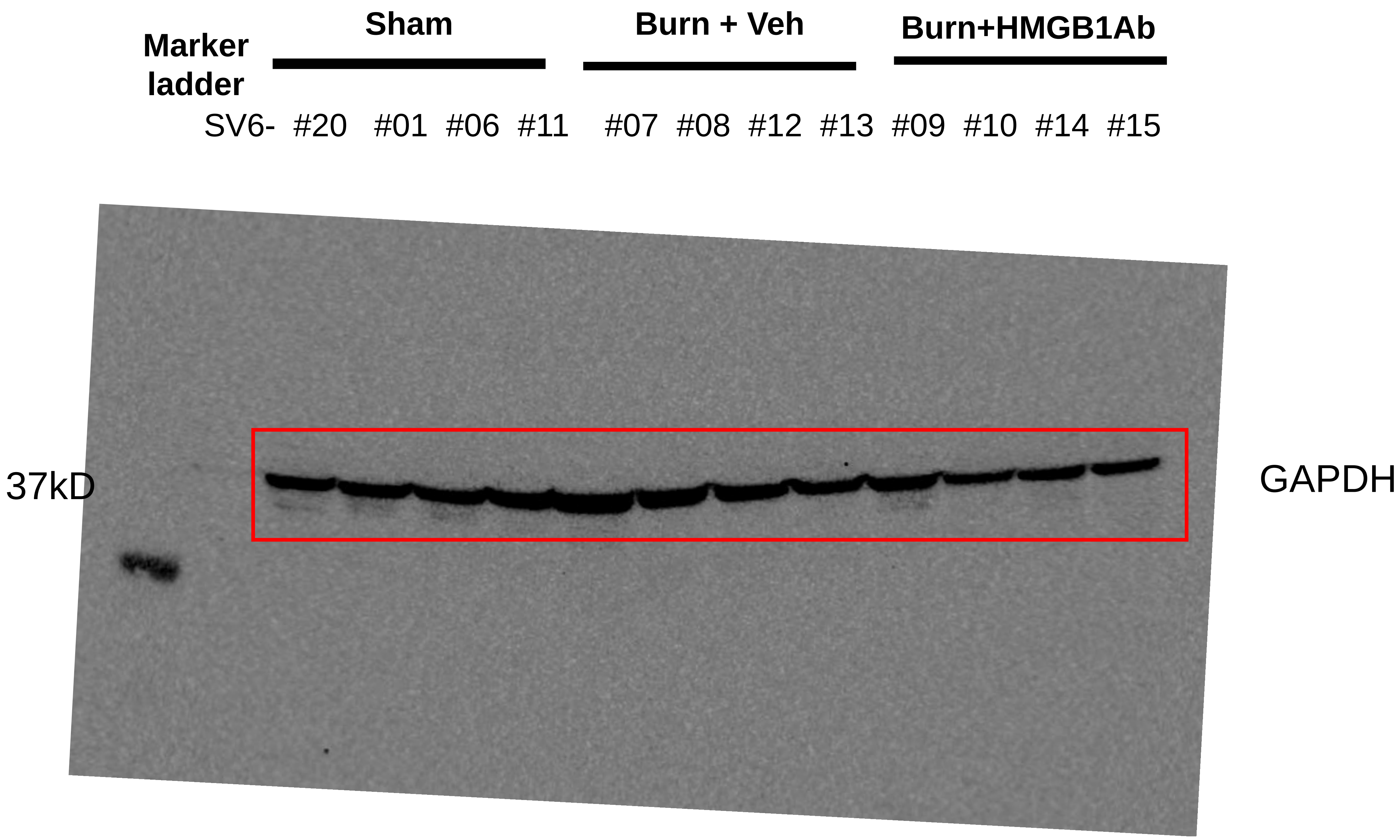

Supplemental figure 2.16 for figure 4h: Western blot raw image of GAPDH expression in muscle tissue from sham burn rats (Sham), or burn rats with vehicle treatment (Burn+Veh) and with HMGB1 antibody treatment (Burn+HMGB1) at day 3 (n=4/each group). The 1st lane is protein marker ladder, following with 12 20µg of protein lysate samples extracted from labeled individual animal in the study (SV6-). A red box circled GAPDH protein bands at the range of 37kD .

[file name: L-h#09-GAPDH-SongJ 2022-04-27 09h15m20s-c-gapdh(Chemiluminescence).tif]
