# Supplementary material for: Acute muscle mass loss was alleviated with HMGB1 neutralizing antibody treatment in severe burned rats
Source: Sci Rep. 2023 Jun 24;13:10250. doi: 10.1038/s41598-023-37476-4 (PMC10290662; doi:10.1038/s41598-023-37476-4)
Supplement: Supplementary file 4 — Supplementary Table S1. [file 41598_2023_37476_MOESM4_ESM.docx]

**Table S1: Flow cytometry antibodies**

| **Marker** | **Color** | **Host/Target** | **Isotype** | **Clone** | **Supplier** | **Cat#** |
| --- | --- | --- | --- | --- | --- | --- |
| TCR γ/δ | VioBlue | cell line α-Rat | IgG1 | REA547 | Miltenyi Biotec | 130‑108‑692 |
| TCR α/β | APC | Mouse α-Rat | IgG1 κ | R73 | BioLegend | 201110 |
| CD4 | BV786 | Mouse α-Rat | IgG2a κ | OX-35 | BD Biosci | 740912 |
| CD80 | BUV395 | Mouse α-Rat | IgG1 κ | 3H5 | BD Biosci | 743869 |
| CD86 | BV510 | Mouse α-Rat | IgG1 κ | 24F | BD Biosci | 743212 |
| CD127 | DyLight 594 | Mouse α-Rat | IgG2a | 717519 | Novus Bio | FAB8484M |
| CD62L | BV650 | Hamster α-Rat | IgG2 λ1 | HRL1 | BD Biosci | 743150 |
| IFNγ | Alexa Fluor 680 | Rabbit α-Rat | IgG | - | Bioss | bs‑0480R‑A680 |
| TNFα | PerCP | Rabbit α-Rat | IgG κ | TNF/1500R | Biotium | BNCP1500‑250 |
| Granulocytes | FITC | Mouse α-Rat | IgM | HIS48 | ThermoFisher | 11‑0570‑82 |
| CD11b | APC | Mouse α-Rat | IgA κ | WT.5 | BD Biosci | 562102 |
| MHC II | PerCP-eFluor 710 | Mouse α-Rat | IgG1 κ | OX17 | Thermo Fisher | 46‑0463‑82 |
| TNFα | PE | Hamster α-Rat | IgG | TN3-19.12 | Thermo Fisher | 12‑7423‑41 |
| IL-1β | DyLight 405 | Mouse α-Rat | IgG1 κ | 43N1G6 | Novus Bio | NBP2‑27342V |

.
