# Supplementary material for: Acute muscle mass loss was alleviated with HMGB1 neutralizing antibody treatment in severe burned rats
Source: Sci Rep. 2023 Jun 24;13:10250. doi: 10.1038/s41598-023-37476-4 (PMC10290662; doi:10.1038/s41598-023-37476-4)
Supplement: Supplementary file 5 — Supplementary Table S2. [file 41598_2023_37476_MOESM5_ESM.docx]

**Table S2: Antibodies used for Western blotting**

| **Antigen** | **Antibody cat # (dilution)** | **Source** |
| --- | --- | --- |
| HMGB1 | 10829-1-AP (1k) | Thermal Fisher |
| C-caspase 3 | 9662 (1k) | Cell Signaling |
| Murf1/2/3 | ab172479 (2k) | Abcam |
| Cytochrome c | 4280 (1k) | Cell Signaling |
| HSP60 | 12165 (1k) | Cell Signaling |
| Myogenin | MA5-11486 (1k) | Thermal Fisher |
| LC3A/B | 12741(1k) | Cell Signaling |
| Ubiquitin | 43124 (1k) | Cell Signaling |
| Beclin-1 | 3495 (1k) | Cell Signaling |
| Atg 12 | 4180(1k) | Cell Signaling |
| Atg 7 | 2631(1k) | Cell Signaling |
| Atg 3 | 3415(1k) | Cell Signaling |
| Atg 5 | 8540(1k) | Cell Signaling |
| PCNA | 13110 (1k) | Cell Signaling |
| GAPDH | 5174 (3k) | Cell Signaling |
