# Supplementary material for: Acute muscle mass loss was alleviated with HMGB1 neutralizing antibody treatment in severe burned rats
Source: Sci Rep. 2023 Jun 24;13:10250. doi: 10.1038/s41598-023-37476-4 (PMC10290662; doi:10.1038/s41598-023-37476-4)
Supplement: Supplementary file 6 — Supplementary Table S3. [file 41598_2023_37476_MOESM6_ESM.docx]

**Table S3. Detailed flow cytometry analysis data**

| **S3A. Immune profile of bone marrow cells in burn rats (± HMGB1 Ab)** | | | | | | | | | | | | | | | | | | | | |
| --- | --- | --- | --- | --- | --- | --- | --- | --- | --- | --- | --- | --- | --- | --- | --- | --- | --- | --- | --- | --- |
| **Pop#** | | **Bone marrow cells** | | | | | **Values** | | | **Sham** | | | **Burn**  **Vehicle** | | | | | **Burn**  **HMGB1 Ab** | | |
| 1 | | CD11b^+^MHCII^-^ | | | | | Mean | | | 1.32 | | | 2.37** | | | | | 1.54^ | | |
|  |  |  |  |  |  |  | ±SEM | | | 0.15 | | | 0.39 | | | | | 0.13 | | |
| 2 | | CD11b^+^MHCII^-^TNFα^+^ | | | | | Mean | | | 1.24 | | | 3.20** | | | | | 1.21^^ | | |
|  |  |  |  |  |  |  | ±SEM | | | 0.24 | | | 0.68 | | | | | 0.31 | | |
| 3 | | CD11b^+^MHCII^-^ IL1β^+^ | | | | | Mean | | | 0.81 | | | 1.75** | | | | | 0.61^^ | | |
|  |  |  |  |  |  |  | ±SEM | | | 0.23 | | | 0.26 | | | | | 0.13 | | |
| 4 | | CD11b^+^MHCII^+^ | | | | | Mean | | | 13.23 | | | 13.26 | | | | | 14.49 | | |
|  |  |  |  |  |  |  | ±SEM | | | 0.78 | | | 2.52 | | | | | 2.07 | | |
| 5 | | CD11b^+^MHCII^+^TNFα^+^ | | | | | Mean | | | 1.12 | | | 2.35* | | | | | 1.37^ | | |
|  |  |  |  |  |  |  | ±SEM | | | 0.44 | | | 0.42 | | | | | 0.29 | | |
| 6 | | CD11b^+^MHCII^+^ IL1β^+^ | | | | | Mean | | | 5.63 | | | 8.32* | | | | | 6.88^ | | |
|  |  |  |  |  |  |  | ±SEM | | | 0.90 | | | 1.48 | | | | | 1.23 | | |
| **S3B. Immune profile of αβTCR^+^ T cells in burn rats (± HMGB1 Ab)** | | | | | | | | | | | | | | | | | | | | |
| **Pop#** | **αβTCR^+^ T cells** | | | **Values** | | **PBMCs** | | | | | | | | **Splenocytes** | | | | | | |
|  |  |  |  |  |  | Sham | | Burn  Vehicle | | | Burn  HMGB1 Ab | | | Sham | | Burn  Vehicle | | | Burn  HMGB1 Ab | |
| 1 | CD4^+^ | | | Mean | | 66.15 | | 73.24*** | | | 73.23*** | | | 60.95 | | 66.84 | | | 66.41 | |
|  |  |  |  | ±SEM | | 1.213 | | 0.83 | | | 1.24 | | | 2.47 | | 2.05 | | | 1.50 | |
| 2 | CD4^+^IFNγ^+^ | | | Mean | | 0.55 | | 1.58* | | | 0.41^^ | | | 0.71 | | 2.24** | | | 0.64^^ | |
|  |  |  |  | ±SEM | | 0.194 | | 0.25 | | | 0.14 | | | 0.19 | | 0.36 | | | 0.14 | |
| 3 | CD4^+^TNFα^+^ | | | Mean | | 0.75 | | 1.21* | | | 0.39^ | | | 2.39 | | 3.15 | | | 1.79^ | |
|  |  |  |  | ±SEM | | 0.29 | | 0.39 | | | 0.09 | | | 0.47 | | 0.75 | | | 0.34 | |
| 4 | CD4^+^CD80^+^ | | | Mean | | 3.79 | | 5.14 | | | 2.81 | | | 6.11 | | 12.48* | | | 6.72^ | |
|  |  |  |  | ±SEM | | 0.85 | | 1.22 | | | 0.79 | | | 1.53 | | 2.67 | | | 1.30 | |
| 5 | CD4^+^CD86^+^ | | | Mean | | 1.85 | | 1.80 | | | 1.01 | | | 2.75 | | 4.34 | | | 2.63 | |
|  |  |  |  | ±SEM | | 0.55 | | 0.46 | | | 0.35 | | | 0.80 | | 0.84 | | | 0.47 | |
| 6 | CD4^+^Teff  CD62L^-^CD127^-^ | | | Mean | | 13.10 | | 16.46 | | | 10.77^ | | | 23.43 | | 34.39** | | | 27.25^ | |
|  |  |  |  | ±SEM | | 2.21 | | 1.64 | | | 1.325 | | | 2.54 | | 2.65 | | | 2.59 | |
| 7 | CD4^+^Tem  CD62L^-^CD127^+^ | | | Mean | | 18.66 | | 15.70 | | | 14.43 | | | 23.02 | | 24.13 | | | 26.29 | |
|  |  |  |  | ±SEM | | 2.89 | | 2.48 | | | 1.94 | | | 3.90 | | 2.33 | | | 2.24 | |
| 8 | CD4^+^Tim  CD62L^+^CD127^-^ | | | Mean | | 21.08 | | 25.93 | | | 23.61 | | | 17.53 | | 16.59 | | | 14.94 | |
|  |  |  |  | ±SEM | | 1.08 | | 1.48 | | | 1.58 | | | 2.04 | | 2.39 | | | 1.34 | |
| 9 | CD4^+^Tn­_Tcm  CD62L^+^CD127^+^ | | | Mean | | 47.13 | | 41.90 | | | 51.20 | | | 36.05 | | 24.88* | | | 31.53^ | |
|  |  |  |  | ±SEM | | 4.43 | | 1.29 | | | 2.27 | | | 4.72 | | 3.19 | | | 3.28 | |
| **S3C. Immune profile of γδTCR^+^ T cells in burn rats (± HMGB1 Ab)** | | | | | | | | | | | | | | | | | | | | |
| **Pop#** | **γδTCR^+^ T cells** | | **Values** | | **PBMCs** | | | | | | | | | | **Splenocytes** | | | | | |
|  |  |  |  |  | Sham | | | | Burn  Vehicle | | | Burn  HMGB1 Ab | | | Sham | | Burn  Vehicle | | | Burn  HMGB1 Ab |
| 1 | γδTCR^+^ | | Mean | | 1.48 | | | | 1.07 | | | 1.06 | | | 1.52 | | 0.85** | | | 0.89** |
|  |  |  | ±SEM | | 0.18 | | | | 0.18 | | | 0.06 | | | 0.21 | | 0.13 | | | 0.14 |
| 2 | γδTCR^+^IFNγ^+^ | | Mean | | 1.42 | | | | 3.06* | | | 0.49^^^ | | | 0.98 | | 3.36** | | | 1.17^ |
|  |  |  | ±SEM | | 0.41 | | | | 0.63 | | | 0.09 | | | 0.34 | | 0.94 | | | 0.28 |
| 3 | γδTCR^+^TNFα^+^ | | Mean | | 1.31 | | | | 3.28** | | | 1.46^ | | | 3.73 | | 18.02*** | | | 5.49^^ |
|  |  |  | ±SEM | | 0.27 | | | | 0.44 | | | 0.26 | | | 0.65 | | 4.87 | | | 0.77 |
| 4 | γδTCR^+^CD80^+^ | | Mean | | 4.05 | | | | 6.56* | | | 2.71^ | | | 6.038 | | 13.16** | | | 7.35^ |
|  |  |  | ±SEM | | 0.84 | | | | 0.97 | | | 0.51 | | | 0.95 | | 2.87 | | | 1.53 |
| 5 | γδTCR^+^CD86^+^ | | Mean | | 6.23 | | | | 6.33 | | | 1.85^ | | | 19.95 | | 26.06* | | | 14.17^^ |
|  |  |  | ±SEM | | 2.61 | | | | 1.80 | | | 0.41 | | | 5.49 | | 5.23 | | | 1.54 |
| 6 | γδTCR^+^Teff  CD62L^-^CD127^-^ | | Mean | | 21.77 | | | | 33.11* | | | 18.67^ | | | 25.367 | | 40.96** | | | 32.5 |
|  |  |  | ±SEM | | 4.46 | | | | 7.17 | | | 2.93 | | | 4.34 | | 3.20 | | | 2.14 |
| 7 | γδTCR^+^Tem  CD62L^-^CD127^+^ | | Mean | | 11.84 | | | | 5.91 | | | 11.11 | | | 20.67 | | 15.76 | | | 13.55 |
|  |  |  | ±SEM | | 1.60 | | | | 1.46 | | | 3.94 | | | 2.42 | | 1.39 | | | 1.23 |
| 8 | γδTCR^+^Tim  CD62L^+^CD127^-^ | | Mean | | 34.57 | | | | 45.64 | | | 46.91 | | | 23.45 | | 22.37 | | | 25.91 |
|  |  |  | ±SEM | | 4.31 | | | | 6.67 | | | 7.49 | | | 3.39 | | 4.53 | | | 3.76 |
| 9 | γδTCR^+^Tn­_Tcm  CD62L^+^CD127^+^ | | Mean | | 31.82 | | | | 15.33 | | | 23.31 | | | 30.50 | | 20.91 | | | 28.01 |
|  |  |  | ±SEM | | 2.93 | | | | 2.47 | | | 3.53 | | | 5.19 | | 1.58 | | | 3.66 |

Sprague Dawley rats were subjected to sham burn or 30% TBSA burn, treated with one dose of vehicle or anti-HMGB1 antibody and euthanized 3 days later. Single cell suspensions of bone marrow cells, peripheral blood mononuclear cells (PBMCs), and splenocytes were labeled with fluorescent-conjugated antibodies and analyzed by flow cytometry. Data were tested by D’Agostino & Pearson Omnibus Normality test. Datasets were analyzed by 1-way analysis of variance (ANOVA) followed by Tukey’s post-hoc test or Kruskal-Wallis H/Dunn’s post-hoc test. Data are presented as mean values ± SEM and derived from n ≥ 6 rats per group. Significance is annotated with * Sham vs Burn + Vehicle or Burn + HMGB1 Ab and ^ Burn + Vehicle vs Burn + HMGB1 Ab, and p values of <0.05, <0.01, and <0.001 are presented with one, two, and three symbols, respectively
